# Profiling the neuroimmune cascade in 3xTg-AD mice exposed to successive mild traumatic brain injuries

**DOI:** 10.1186/s12974-024-03128-1

**Published:** 2024-06-13

**Authors:** Alyssa F. Pybus, Sara Bitarafan, Rowan O. Brothers, Alivia Rohrer, Arushi Khaitan, Felix Rivera Moctezuma, Kareena Udeshi, Brae Davies, Sydney Triplett, Martin N. Griffin, Eric B. Dammer, Srikant Rangaraju, Erin M. Buckley, Levi B. Wood

**Affiliations:** 1 Wallace H. Coulter Department of Biomedical Engineering, Georgia Institute of Technology and Emory University, Atlanta, GA USA; 2 George W. Woodruff School of Mechanical Engineering, Georgia Institute of Technology, Atlanta, GA USA; 3 School of Biological Sciences, Georgia Institute of Technology, Atlanta, GA USA; 4 Center for Neurodegenerative Diseases, School of Medicine, Emory University, Atlanta, GA USA; 5 Department of Neurology, School of Medicine, Yale University, New Haven, CT USA; 6 Department of Pediatrics, School of Medicine, Emory University, Atlanta, GA USA; 7 Children’s Healthcare of Atlanta, Atlanta, GA USA; 8 Parker H. Petit Institute for Bioengineering and Bioscience, Georgia Institute of Technology, Atlanta, GA USA

**Keywords:** Traumatic brain injury, Mild traumatic brain injury, Alzheimer’s pathology, Cytokines, Neuroinflammation, MAPK, 3xTg-AD, Astrocyte reactivity

## Abstract

Repetitive mild traumatic brain injuries (rmTBI) sustained within a window of vulnerability can result in long term cognitive deficits, depression, and eventual neurodegeneration associated with tau pathology, amyloid beta (Aβ) plaques, gliosis, and neuronal and functional loss. However, a comprehensive study relating acute changes in immune signaling and glial reactivity to neuronal changes and pathological markers after single and repetitive mTBIs is currently lacking. In the current study, we addressed the question of how repeated injuries affect the brain neuroimmune response in the acute phase of injury (< 24 h) by exposing the 3xTg-AD mouse model of tau and Aβ pathology to successive (1x-5x) once-daily weight drop closed-head injuries and quantifying immune markers, pathological markers, and transcriptional profiles at 30 min, 4 h, and 24 h after each injury. We used young adult 2–4 month old 3xTg-AD mice to model the effects of rmTBI in the absence of significant tau and Aβ pathology. We identified pronounced sexual dimorphism in this model, with females eliciting more diverse changes after injury compared to males. Specifically, females showed: (1) a single injury caused a decrease in neuron-enriched genes inversely correlated with inflammatory protein expression and an increase in AD-related genes within 24 h, (2) each injury significantly increased a group of cortical cytokines (IL-1α, IL-1β, IL-2, IL-9, IL-13, IL-17, KC) and MAPK phospho-proteins (phospho-Atf2, phospho-Mek1), several of which co-labeled with neurons and correlated with phospho-tau, and (3) repetitive injury caused increased expression of genes associated with astrocyte reactivity and macrophage-associated immune function. Collectively our data suggest that neurons respond to a single injury within 24 h, while other cell types, including astrocytes, transition to inflammatory phenotypes within days of repetitive injury.

**Supplementary Information:**

The online version contains supplementary material available at 10.1186/s12974-024-03128-1.

## Introduction

Traumatic brain injury (TBI) results in approximately 2.5 million emergency department visits every year in the Unites States alone. Approximately 75% of TBI cases are classified as mild (mTBI), and these mild cases carry an annual cost burden of $17 billion [1, 2]. An estimated 10–40% of mTBIs are associated with persistent functional impairment lasting longer than one month and in some cases up to a year [3–6]. Moreover, repeated mTBIs (rmTBIs) sustained within a window of vulnerability can intensify pathological and functional consequences [7, 8]. These repeated injuries, which are often observed among athletes in high contact sports such as American football and boxing, can result in the development of Alzheimer’s disease-like pathology, including neurofibrillary tangles and amyloid beta (Aβ) plaques [9]. Current treatment paradigms for (r)mTBI are severely lacking and focus on the alleviation of symptoms, rather than targeting the underlying mechanisms of injury. There is thus an urgent need to illuminate the post-injury neuro-molecular sequelae for development of targeted therapies.

Mounting evidence suggests the involvement of brain immune signaling as a key driver of long-term outcome following mTBI [10–21]. Brain immune signaling is implicated in several neurodegenerative diseases associated with mTBI, including Alzheimer’s disease (AD) [22–24] and Parkinson’s disease [25–27]. Moreover, the roles of immune signaling in driving pathology following severe TBI has been relatively well-studied [28–32], and preliminary evidence shows similar patterns in (r)mTBI [28, 33–37]. Indeed, prior research from our own group has identified correlations between lower cerebral blood flow (a potential biomarker of worse cognitive outcome) and increased mitogen-activated protein kinase (MAPK) signaling, cytokine expression, and microglial activation after repetitive closed-head injuries (CHI) in wild type mice, supporting an essential relationship between brain immune signaling and outcome after mTBI [38]. However, a comprehensive study relating acute changes in immune signaling and glial activation to neuronal changes and pathological markers after single and repetitive mTBIs is currently lacking and would provide a much-needed perspective to guide the search for new therapies.

The acute pathophysiology of mTBI has previously been defined by the “neurometabolic cascade of concussion,” in which neurons undergo axonal injury and dysfunction, altered neurotransmission, ionic flux, and indiscriminate release of glutamate, while many brain cells undergo a hyperglycolysis energy crisis to restore homeostasis [39, 40]. When Drs. Giza and Hovda reviewed the neurometabolic cascade in 2014 [40], relatively little was known about mTBI-induced inflammatory changes, but research over the last decade has more fully defined changes in immune signaling, microglial activation, and astrocyte reactivity, as well as their effects on secondary injury after TBI [28, 41–44]. For example, recent studies suggest that astrocytes rapidly respond to mechanical stress after injury, potentially via opening of mechanosensitive ion channels, leading to activation of MAPK signaling, ATP release, and reactive astrogliosis [41, 45–51]. Here we propose to similarly define the acute “neuroimmune cascade” of mTBI. These clear changes in microglial and astrocyte responses post-mTBI suggest a broad and complex neuroimmune response to mTBI. Thus, a comprehensive understanding of neuroimmune signaling and its hypothesized relationship to neuronal, glial, and pathogenic changes after injury is critical to identifying drivers of secondary injury as well as determining therapeutic targets to mitigate adverse cognitive and pathological outcomes.

**Fig. 1:**
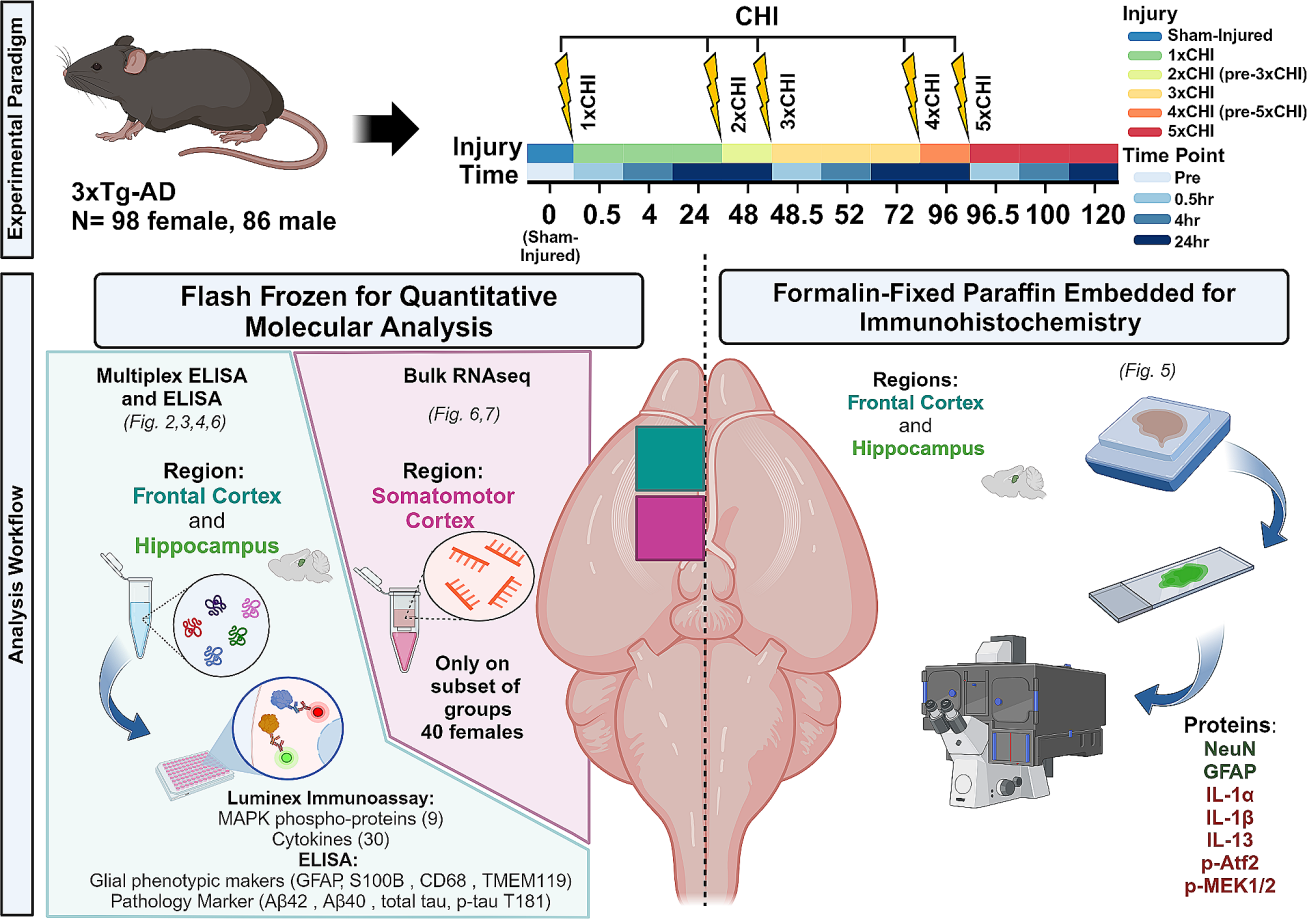
Experimental design. Male and female 3xTg-AD mice were exposed to 1x-5xCHIs and brain tissues were collected at multiple timepoints after each injury. Left hemispheres were micro-dissected for quantitative protein and mRNA analysis. Right hemispheres were used for immunohistochemistry. Main text figures associated with each experimental outcome are indicated in italic font

In the current study, we sought to define the neuroimmune cascade of (r)mTBI by profiling protein and transcriptional changes associated with immune signaling, pathology, and the neuronal, microglial, and astrocytic compartments (Fig. 1). We specifically hypothesized that (r)mTBI would drive brain immune signaling in a manner correlated with markers of neurodegeneration, such as tau, Aβ, astrocyte reactivity, and microglial activation. Because we aimed to identify relationships between immune signaling and molecular markers associated with AD-related pathology, and wild-type mice show inconsistent evidence of pathological changes after TBI [52–54], we used the triple transgenic model of Alzheimer’s-like pathology (3xTg-AD), which contains human mutant forms of genes in the Aβ processing pathway (*APP*, *PSEN1*) and tau (*MAPT*). This model is widely used to study AD and is increasingly utilized in brain injury research [55–62]. Here, we sought to concomitantly define the effects of successively increasing mild traumatic brain injuries on the following molecular outcomes in the frontal cortex and hippocampus: (1) the kinetics of brain immune signaling and cytokine expression, (2) tau phosphorylation and Aβ burden, (3) markers of astrocyte reactivity and microglial activation, and (4) transcriptional profiling of the somatomotor cortex (females only) (Fig. 1). Our analyses revealed pronounced effects of repetitive injury on all molecular outcomes. Further, the inclusion of both male and female 3xTg-AD mice in our study revealed strong effects of sexual dimorphism in this model across all outcomes, with males showing higher basal levels of immune signaling but females showing more pronounced changes in response to injury.

## Materials and methods

All resources with available Research Resource Identifiers (RRIDs) are listed in Table S1.

### Study protocol

3xTg-AD on C57BL/6J mice (Jackson Laboratory, strain 033930) were aged 2–4 months (98 females, 86 males). All protocols were approved by the U.S. Army Medical Research and Development Command Animal Care and Use Review Office (ACURO) and the Emory University Institutional Animal Care and Use Committee (IACUC) in accordance with National Institutes of Health and ARRIVE guidelines. Mice were housed in a pathogen free facility with a twelve-hour light/dark cycle. Food and water were provided *ad libitum*. Mice were randomly assigned to one of three injury groups: one closed head injury (1xCHI), three closed-head injuries spaced once daily (3xCHI), and five closed-head injuries spaced once daily (5xCHI). Sham-injured controls were exposed to the same numbers of anesthesia exposures as each injured group but were not exposed to injury. As noted below, sham-injured groups were combined to reduce animal numbers. Animals were euthanized by cervical dislocation under 4.5% isoflurane (1 L/min, 100% oxygen), and the brain was harvested for molecular and pathological assessment at the following time points for each group: pre-injury, 30 min, 4 h, and 24 h post-injury. Left hemispheres were micro-dissected into frontal cortex (approximately bregma + 1 mm to + 2.5 mm), hippocampus, and somatomotor cortex (approximately bregma − 1 mm to + 1 mm) sections, flash-frozen by submerging sample tubes in liquid nitrogen, then stored at -80 °C for follow-on molecular analysis. Right hemispheres were drop-fixed in 4% paraformaldehyde then processed and embedded in paraffin for immunohistochemistry analysis. Sample sizes (*n* = 6–8 per injury time point group in protein data, *n* = 2–4 in RNAseq data) are provided in Table S2. We powered the current study with *n* = 12 (6 males, 6 females) per time point and injury based on our prior cytokine data from wild-type mice exposed to 5xCHI (α = 0.05, 80% power). Having found that males and females exhibited distinct baseline and injury responses to many of our molecular markers, we separated them in the current study.

### Closed head injury model

This work was conducted using a previously characterized weight drop closed-head injury (CHI) model [63, 64]. Animals were anesthetized using 4.5% isoflurane (1 L/min, 100% oxygen) prior to injury. The injury was delivered by dropping a 54 g bolt down a 0.96 m vertical guide tube (49035K85, McMaster-Carr, Elmhurst IL) onto the dorsal aspect of the head (skull intact), targeting between the approximate location of the coronal and lambdoid structures. The anesthetized animal was positioned under the guide tube on a Kimwipes® task wipe (Kimberly-Clark, Irving, TX). The animal was grasped by the base of the tail so that on impact the mouse penetrated the task wipe and underwent rapid, unrestricted rotation of the head in the anterior-posterior plane. Following injury, animals were monitored continuously until they regain righting reflex. They were then checked every 15 min for the first hour, then daily. This diffuse injury model is not expected to cause skull fracture or focal injury [65] and we did not observe skull fracture in any injured animals. Sham-injured mice were age- and sex- matched and received the same exposures to anesthesia, matched to all injury groups and time points, but were not subject to closed-head injury. Because principal component analysis did not identify significant differences in our protein markers between sham-injured animals of distinct time points and numbers of exposures to anesthesia, we treated all sham-injured animals of the same sex as a single experimental group.

### Quantification of immune and pathological proteins

To comprehensively profile the immune signaling kinetics after repeated CHI in 3xTg-AD male and female mice, we quantified four classes of proteins: glial phenotypic markers (astrocyte reactivity markers GFAP and S100B; microglial activation marker CD68 and homeostatic marker TMEM119), molecular markers of pathology (Aβ40, Aβ42, total tau, and phospho-tau T181), nine MAPK phospho-proteins, and 30 cytokines (in total 47 measured proteins, plus ratios Aβ42/40 and pTau/tTau). We collected frontal cortex and hippocampus samples from the left hemisphere of the three injury groups (1xCHI, 3xCHI, and 5xCHI) across four time points each (pre-injury and 30 min, 4 h, 24 h post-injury), where the pre-injury 1xCHI group represents sham-injured controls. In total, data were collected from 98 female mice and 86 male mice. Two female and three male mice were excluded from our analysis after Mahalanobis outlier detection (α < 0.001) of all protein measurements, leaving *n* = 96 females and *n* = 83 males.

Cortical and hippocampal brain tissue sections were lysed using the Bio-Plex cell lysis kit (Bio-Rad Laboratories #171,304,011) and protein concentrations were determined using a Pierce BCA Protein Assay (Thermo Fisher #23,225). Multiplexed cytokine quantification was conducted using the Milliplex® MAP Mouse Cytokine/Chemokine 32-Plex kit (Eotaxin, G-CSF, GM-CSF, IFN-γ, IL-1α, IL-1β, IL-2, IL-3, IL-4, IL-5, IL-6, IL-7, IL-9, IL-10, IL-12p40, IL-12p70, IL-13, IL-15, IL-17, IP-10, KC, LIF, LIX, MCP-1, M-CSF, MIG, MIP-1α*, MIP-1β, MIP-2, RANTES, TNF-α, and VEGF*) (Millipore Sigma MCYTMAG-70 K-PX32). Cytokines marked with an asterisk did not fall within a linear range of bead fluorescent intensity vs. protein concentration and were not included in our analysis. Multiplexed phospho-protein quantification was conducted for the MAPK signaling pathway using the Bio-Plex Pro™ Cell Signaling MAPK Panel 9-plex kit (phospho-Atf2 T71, phospho-Erk1/2 T202/Y204 T185/Y187, phospho-HSP27 S78, phospho-JNK T183/Y185, phospho-Mek1 S217/S221, phospho-p38 MAPK T180/Y182, phospho-p53 S15, phospho-p90 RSK S380, phospho-Stat3 S727) (Bio-Rad Laboratories LQ00000S6KL81S). Prior to analysis, lysates were thawed on ice and centrifuged at 4 °C for 10 min at 15,500 g. Protein concentrations were normalized with Milliplex® MAP Assay Buffer (EMD Millipore, Billerica, MA) to 6 µg protein per 12.5 µL for cytokine analysis and 1 µg protein per 12.5 µL for the MAPK pathway analysis. These protein concentrations were selected because they fell within the linear range of bead fluorescent intensity vs. protein concentration for detectable analytes. Quantification of pathological markers was conducted for total tau, phospho-tau (T181), Aβ 1–40, and Aβ 1–42 using the Milliplex® MAP Human Amyloid Beta and Tau Multiplex Assay kit (Millipore Sigma HNABTMAG-68 K). All kits were read on a MAGPIX® system (Luminex, Austin, TX).

We quantified astrocyte reactivity markers glial fibrillary acidic protein (GFAP) and S100 calcium binding protein B (S100B) as well as macrophage and microglia activation marker cluster of differentiation 68 (CD68) and microglial homeostatic marker transmembrane protein 119 (TMEM119) via enzyme-linked immunosorbent assay (ELISA). Protein concentrations were normalized with respective assay diluents from each kit to 2.5 µg/ml in 50 µL for the GFAP ELISA kit (Abcam ab233621), 50 µg/ml in 50 µL for the S100B ELISA kit (Abcam ab234573), and 0.1 µg/µL in 50ul for the CD68 and TMEM119 ELISA kits (Lifespan Biosciences LS-F11095 and LS-F52734). All loaded protein concentrations were selected because they fell within the linear range of absorbance vs. protein concentration for detectable analytes. Due to the large number of samples and plate-to-plate variation observed in immuno-assays [66], we ran single technical replicates for each sample. For each ELISA kit, we validated its ability to report protein levels by cross-validating against quantitative analysis of Western blot for each analyte (*n* = 6 samples, anti-GFAP, anti-TMEM119, anti-CD68, SB100B), and by conducting a linear ranging analysis to identify a range of sample loaded that leads to a linearly related output signal [64].

### Adjustment for batch effect and pathology

Male and female 3xTg-AD data were each collected simultaneously for S100B, CD68, and TMEM119 by ELISA, but separated into different assay batches for all other protein measurements. Eight female sample replicates were included in each male batch assay (Luminex and ELISA) and then used to transform the male data into the female space via a linear model for each protein. Computations were conducted using the lm() function in R.

To adjust data for the effects of pathology, linear models were calculated using neuroimmune proteins as dependent variables with the following combinations of independent variables: total tau and phospho-tau T181; Aβ40 and Aβ42; or total tau, phospho-tau T181, Aβ40, and Aβ42. Residuals of each linear model were defined as the pathology-adjusted (tau-adjusted, Aβ-adjusted, or total pathology-adjusted) neuroimmune protein data. Computations were conducted using the lm() function in R.

### Immunohistochemistry

Immunohistochemistry was performed to investigate GFAP, NeuN, IL-1α, IL-1β, IL-13, KC, phospho-Atf2 (T71), phospho-Mek1/2 (S217/S221), and phospho-tau (S262/T263). Right brain hemispheres from each animal were fixed in 4% paraformaldehyde then processed and embedded in paraffin. Tissue slices were cut into 10 μm thick sagittal sections using a rotary microtome (Thermo Fisher) and affixed onto glass microscopy slides (Electron Microscopy Sciences, Hatfield, PA). Tissue slices were deparafinized in xylenes and rehydrated by washing with 100% ethanol, 95% ethanol, and deionized water. Antigen retrieval was performed in a microwave by boiling slides in 10mM sodium citrate buffer at pH 6.0. Slides were rinsed in tris-buffered saline with 0.01% tween (TBST). A hydrophobic ring was drawn around each individual tissue slice using an immunohistochemistry PAP pen (Enzo Life Sciences, Farmingdale, NY), after which samples were blocked for 2 h in blocking buffer, 5% w/v Bovine Serum Albumin (BSA) or 50% goat serum (Sigma-Aldrich) in TBST. Samples were incubated at 4 °C overnight with primary antibodies diluted in blocking buffer: GFAP (1:100, Diagnostic Biosystems Mob064), NeuN (1:200, Abcam ab104224), IL-1α (1:50, Thermo Fisher PA5-89037), IL-1β (1:500, Abcam ab283818), IL-13 (1:100, Abcam ab106732), KC (1:50, Thermo Fisher PA5-86508), phospho-Atf2 T71 (1:50, Thermo Fisher PA5-97332), phospho-Mek1/2 S217/S221 (1:50, Thermo Fisher PA5-105777), or phospho-tau S262/T263 (1:100, Abcam ab92627), as appropriate. Slides were rinsed again in TBST and incubated with either Alexa Fluor 555 and/or Alexa Fluor 488 secondary antibodies (Thermo Fisher) diluted 1:200 in blocking buffer. Slides were counterstained with 1 µg/mL DAPI, rinsed in water, and mounted with VECTASHIELD Antifade Mounting Medium (Vector Laboratories, Burlingame, CA). Samples were imaged using epifluorescent microscopy on a Zeiss Axio Observer Z.1 inverted microscope with a 20x lens and halogen bulb illumination using Zeiss filter set 49 to image DAPI, Zeiss filter set 50 to image Alex flour 647, and Zeiss filter set 20 to image Alexa flour 555. Images were processed using Zen Blue V3.3.

### Data analysis and visualization

Data was analyzed and figures were generated in RStudio (Boston, MA) using the R programming language. Software packages and functions described in this section are denoted by Courier New font. Data processing was conducted using the tidyverse collection of packages [67]. Heatmaps were generated using the R package heatmap3 [68], bar graphs and regression plots were created using the packages ggplot2 [69] and ggpubr [70], and gene set variation analysis was conducted using the gsva package [71] available on Bioconductor. For each molecular assay, we collected background measurements using assay buffer in the absence of biological samples. We subtracted average background measurements from each sample measurement and set negative values to zero. Clustering was conducted using the hclust function of the stats package in R using Euclidean distance in the unweighted pair group method with arithmetic mean. Outlier detection was conducted in R by calculating Mahalanobis distance of each point from the data’s centroid within each grouping of sex and region (female frontal cortex, female hippocampus, male frontal cortex, and male hippocampus) using the ClassDiscovery [72] package. Two female and three male hippocampus samples were outside the cutoff threshold of α = 0.001 and protein data from those animals was discarded in both the frontal cortex and hippocampus sets.

### Bulk tissue RNA sequencing and analysis

We conducted RNA sequencing on a representative subset of 40 3xTg-AD female left somatomotor cortex samples selected from the original 96 for bulk tissue RNA sequencing (available on the NCBI Gene Expression Omnibus under accession number GSE226838). One sample was removed post-sequencing after identification as an outlier animal within the protein data. RNA was extracted using the miRNeasy Micro kit (Qiagen #217,084). Extracted RNA was sent to Admera Health, LLC (South Plainfield, NJ) for sequencing, alignment, and calculation of the count matrix. The count matrix yielded 32,807 non-zero features. Features with fewer than 10 counts in more than four samples (∼ 10%) were filtered out of the analysis (17,844 features). The remaining 14,963 features were normalized by ratio of median method then variance-stabilized in R using the DESeq2 package, available on Bioconductor [73].

Weighted gene co-expression network analysis (WGCNA) was conducted in R using the WGCNA package [74] available on the Comprehensive R Archive Network (CRAN). A WGCNA threshold power of 4 was chosen since it was the smallest threshold that resulted in a scale-free R^2^ value greater than 0.80. We constructed our network in a single block using blockwiseModules() with the following parameters: threshold power of 4, signed modules, a minimum module size of 20, a merge cut height of 0.07, a reassignment threshold of 0.05, a “bicor” correlation type, and a “mean” TOM denominator. Significance of module eigengene (ME) expression between sham-injured and injured samples was assessed by permutation test in which ME values for both populations were randomly re-assigned to either group then permutated mean difference was compared to the true mean difference across 10,000 iterations. Original sample sizes were maintained. Groups were considered significantly different if the true mean difference was greater than or equal to the permutated mean difference in at least 95% of iterations.

Gene ontology was conducted for each of the 14 resulting modules using PANTHER overrepresentation test on PANTHER 17.0, available on the Gene Ontology Resource. We used the mm10 GO biological process complete annotation set with Fisher’s exact test and false discovery rate (FDR) corrected p-values. The test was applied using all genes with a network module membership coefficient (kME) of at least 0.60 and a background of the 14,963 features used to construct the network.

Cell type enrichment was conducted using protocol as published in [75–77]. Briefly, a cell type marker list was created for neurons, oligodendrocytes, endothelia, astrocytes, and microglia from cell type specific mouse brain proteome and transcriptome studies Sharma et al. [78] and Zhang et al. [79]. Fisher exact tests with FDR correction were performed to determine enrichment for each cell type marker list within each WGCNA network module. We used R scripts by Eric Dammer and Divya Nandakumar (Emory University School of Medicine) freely available on GitHub at https://github.com/edammer/CellTypeFET.

Multi-marker Analysis of GenoMic Annotation (MAGMA) [80] was conducted to identify modules with significant enrichment for genes related to Alzheimer’s disease (AD) as identified in genome-wide association studies (GWAS) [81–83]. To implement MAGMA, we used R scripts by Eric Dammer as used in Seyfried et al. [77], freely available on GitHub at https://github.com/edammer/MAGMA.SPA.

Gene set variation analysis (GSVA) was conducted on the normalized and variance-stabilized count matrix in R using the gsva package, also available on Bioconductor [71]. Gene sets used for GSVA included the C2 curated gene sets collection from the Molecular Signatures Database [84, 85] as well as our previously published astrocyte-enriched gene sets [86].

### Figure preparation

Figure 1 was prepared using BioRender.com. Other figures were prepared in Inkscape.

## Results

### Immune, glial, and pathological markers differed between male and female 3xTg-AD mice

**Fig. 2:**
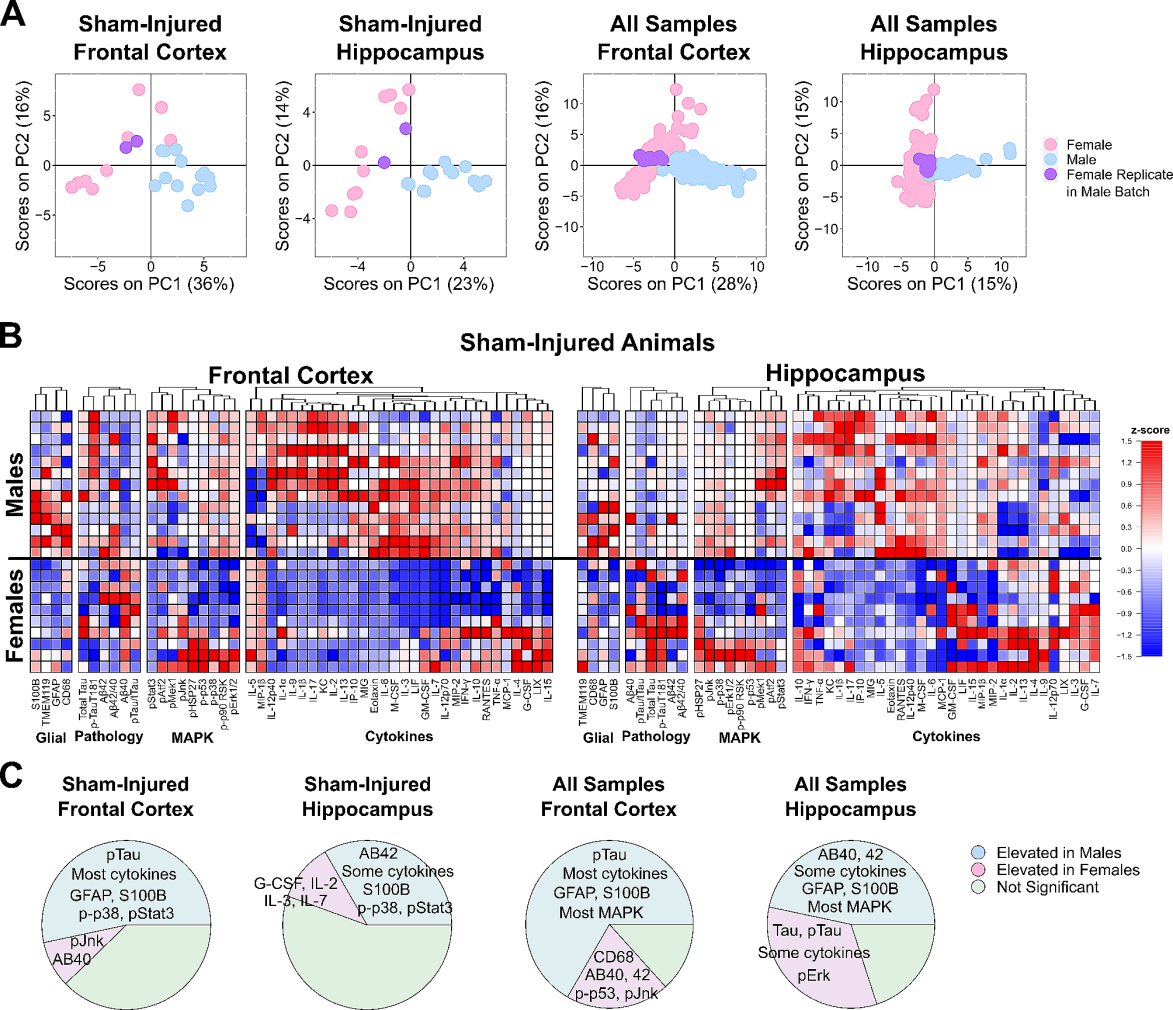
Pronounced sexual dimorphism of immune signaling, glial markers, and molecular markers of pathology in 3xTg-AD mice. **A**) Principal component analysis of combined protein data across only sham-injured animals (left) and all samples (injured and sham-injured, right), reveals strong separation between male and female mice on the first two PCs. Female replicate samples measured in male batch assays are shown in purple, emphasizing that the effects of sex were biological, rather than technical. Percent variance described by each PC is annotated in parentheses. **B**) Sham-injured control mice show distinct baseline sex differences in expression of glial markers, pathological markers, MAPK signaling, and cytokines. Proteins are categorized by function, then clustered by Euclidean distance within each category. Red to blue indicates relatively high to low expression among all sham-injured animals (*n* = 13 males, *n* = 10 females, z-scored). Of the 46 measured immune and pathological marker proteins in sham-injured animals, 37 from the frontal cortex (80%) and 30 from the hippocampus (65%) had higher average expression in males compared to females. **C**) Pie charts for each region and sample set. Left: just sham-injured. Right: all samples, injured and sham-injured show high proportions of all analytes measured (4 glial markers, 4 pathological markers, 9 MAPK, 30 cytokines) that are significantly elevated (*p* < 0.05, Wilcoxon rank-sum) in males (blue) or females (pink). See summary of significant differences in Table S3

Because prior reports showed pronounced differences in pathological burden between male and female 3xTg-AD mice [87, 88], we began the current study by evaluating sex differences among all protein measures: cytokines, MAPK phospho-proteins, glial markers, phosphorylated and total tau, and Aβ40/42. We first obtained a holistic view of variation within the data using a principal component analysis (PCA), which revealed a pronounced effect of sex within both frontal cortex and hippocampus brain regions (Fig. 2A).

To define the specific effects of sex versus injury and time point, we conducted a multiple linear regression of all quantified proteins and phospho-proteins using sex, injury number, and time point as independent variables. This analysis revealed that sex, as compared to all other variables in the model, was the most significant effect (Fig. S1). We next conducted comparisons between sex for each measured protein in (1) sham-injured animals only (Fig. 2B) and (2) all sham-injured and injured animals (Fig. S1A). All proteins determined to be significantly different between sexes based on the sham-injured animals alone were also significantly different between sexes when all samples were used, regardless of injury condition (Fig. 2C, Fig. S2, Table S3).

Female 3xTg-AD samples showed significantly higher levels of Aβ42 in the frontal cortex compared to males (*p* = 0.037, Wilcoxon rank-sum), and higher total and phosphorylated tau at a pathologically relevant residue (T181) [89] in the hippocampus compared to male samples (*p* < 0.0001, Wilcoxon rank-sum; Fig. 2C, Table S3). This result matches most prior studies in 3xTg-AD mice, as reviewed in [87]. Interestingly, we found significantly higher phospho-tau T181 in male compared to female frontal cortices (*p* < 0.0001, Wilcoxon rank-sum; Fig. 2C, Table S3).

In addition to Aβ42 and phospho-tau T181, the majority of the 46 measured immune and pathological marker proteins in both cortical and hippocampal brain regions were elevated on average in males compared to females (Fig. 2B, Fig S1A). Of those, 31 from the frontal cortex (24 in sham-injured only) and 21 from the hippocampus (15 in sham-injured only) reached statistical significance (*p* < 0.05, Wilcoxon rank-sum; Fig. 2C, Fig. S2, Table S3). These included, the astrocyte reactivity markers GFAP and S100B, which were significantly elevated in both the frontal cortex hippocampus compared to females. Taken together, these data suggest an elevated immune signaling baseline in young (2-4mo) male 3xTg-AD mice compared to their age-matched female counterparts.

To determine if differences in immune markers between male and female 3xTg-AD mice could be explained by the well-documented disparities in Aβ and tau in this model [87], we adjusted our data for effects related to Aβ (Aβ40 and Aβ42), tau (total tau and phospho-tau T181), or both (Methods, Fig. S3, Table S4). Adjustment for all pathological markers in the frontal cortex reduced the number of differentially expressed proteins from 31 to 15 elevated in males and from 9 to 3 elevated in females, suggesting that approximately half of all differentially expressed proteins can be explained by pathology. A similar effect is seen in the hippocampus after adjustment: from 21 to 12 proteins elevated in males and 16 to 8 in females. Adjustment for only Aβ-related effects (Aβ40 + Aβ42) in the hippocampus accounted for just 2 differentially expressed proteins (Table S4). The persistence of sexual dimorphism among immune markers (mostly cytokines) after adjustment for pathological measures suggests inherent differences in baseline immune signaling of young male and female 3xTg-AD mice independent of their stage of pathogenesis. Thus, we analyzed male and female data separately in the following analyses.

### Cytokines and MAPK phospho-proteins are elevated after each closed-head injury

**Fig. 3:**
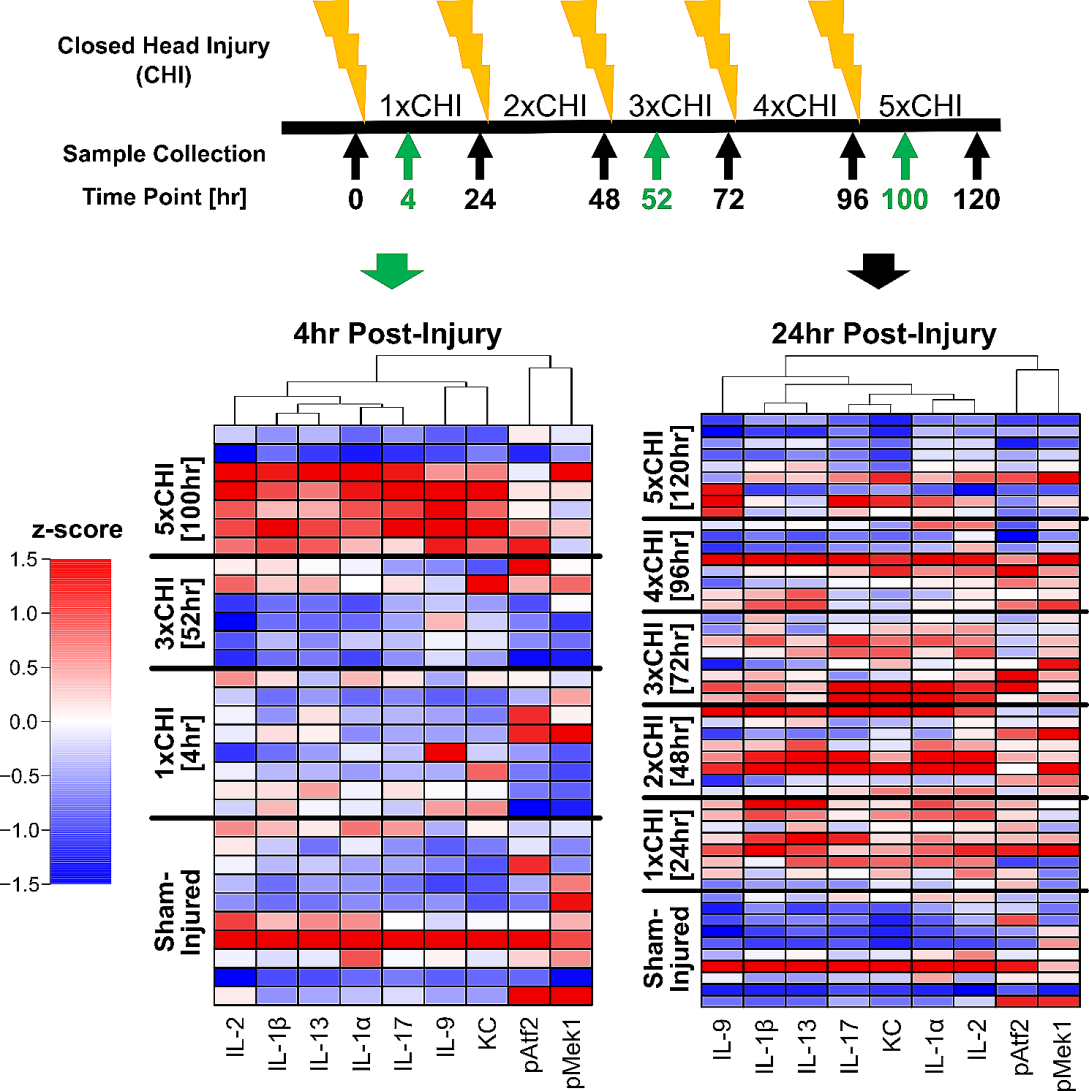
Cytokines and MAPK phospho-proteins in 3xTg-AD females are elevated 4 h after 5xCHI and 24 h after 1x-3x CHI. Hierarchical clustering of cytokines and MAPK phospho-proteins (subset from Fig. S4, “Group 1”) from the frontal cortex of female 2-4mo 3xTg-AD sham-injured mice and 4 h (left) or 24 h (right) after 1x-5x closed head injuries (CHI). Each row represents data from an individual mouse z-scored along each column

To identify patterns of co-varying cytokines and phospho-proteins with injury and time point, we next conducted hierarchical clustering within each sex and brain region (Materials and Methods, Fig. S4-5). We found that 3xTg-AD females exhibited robust variation of signaling proteins with both number of injuries and time point. The clustering identified a group of injury-induced cytokines (IL-9, IL-17, KC, IL-1α, IL-2, IL-1β, IL-13) and MAPK phospho-proteins (phospho-Atf2, phospho-Mek1) that tightly clustered together within the frontal cortex data from female mice (Fig. S4, “Group 1”). Proteins within this group were significantly increased at 24 h after 1x, 2x, and 3xCHI, showed mild increases 24 h after 4xCHI, and returned to baseline sham-injured levels 24 h after 5xCHI (Fig. 3), suggesting a dysregulated immune response after four or more injuries. These proteins were elevated 4 h post-5xCHI compared to sham-injured, 4 h post-1xCHI, and 4 h post-3xCHI animals (Fig. 3), indicating an earlier response after multiple injuries consistent with a “primed” immune state [90]. Although many of the same cytokines and phospho-proteins from Group 1 again clustered together within the 3xTg-AD female hippocampus (Fig. S4) and male 3xTg-AD frontal cortex and hippocampus (Fig. S5), this same trend of an injury-dependent increase was only noted in the female frontal cortex. Together, these data indicate the importance of evaluating multiple time points after injury to fully define the immune response and emphasize the need to evaluate males and females separately.

### Protein and transcript markers of pathology are elevated after injury

**Fig. 4:**
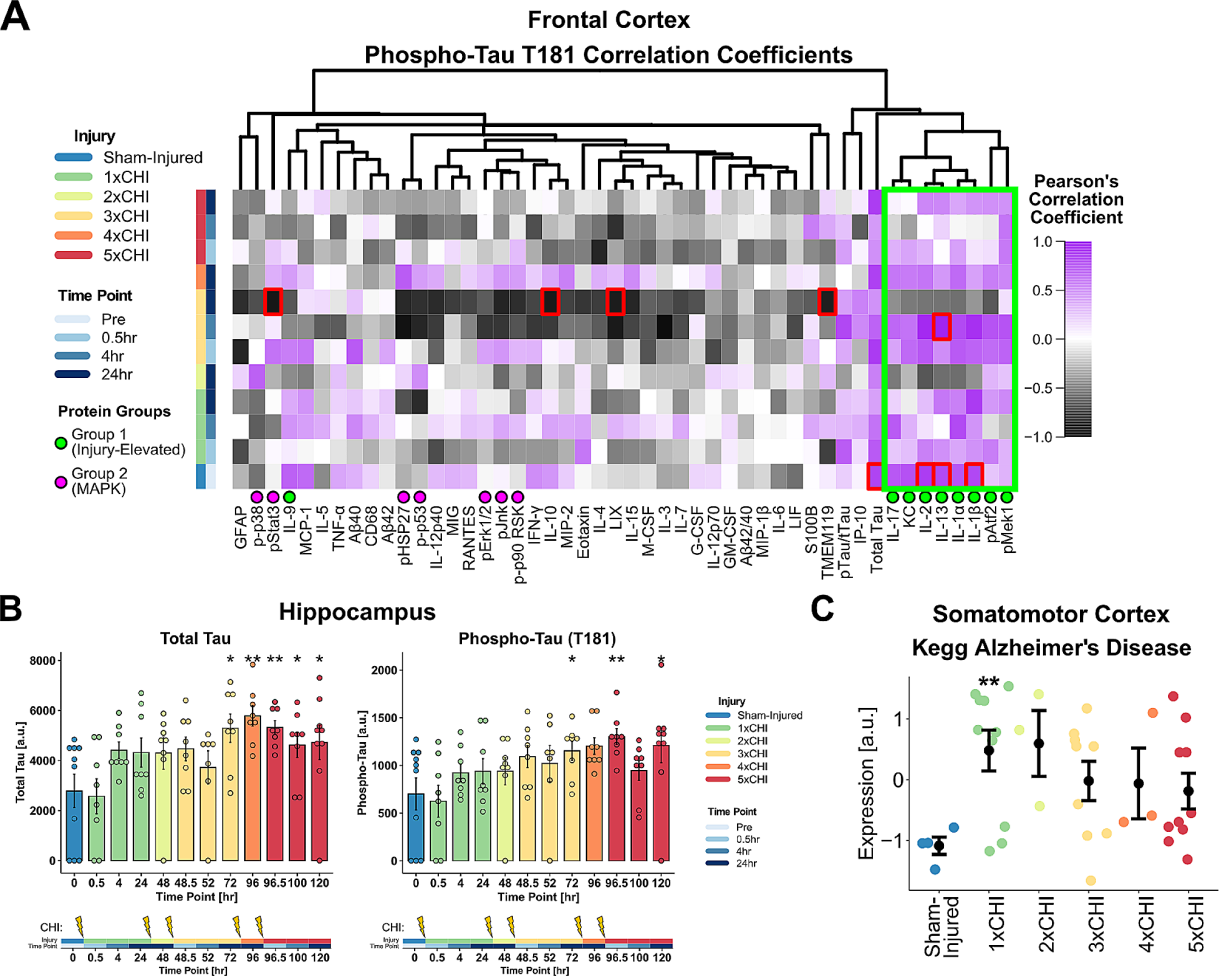
Pathological markers in 3xTg-AD females are increased after injury and associated with cytokines in a region dependent manner. **A**) Heatmap of Pearson’s correlation coefficient of cortical phospho-tau T181 versus each measured cortical protein (columns) within each injury group and time point (rows); red boxes indicate FDR-adjusted *p* < 0.05; green and purple dots represent Group 1 and 2 proteins, respectively. Green box shows eight out of nine Group 1 proteins cluster together and show correlation coefficients *R* > 0.50 with phospho-tau T181. **B**) Total tau is significantly upregulated in the hippocampus at 24 h post-3xCHI and each successive time point compared to sham-injured animals (*p* = 0.014, *p* = 0.0021, *p* = 0.0021, *p* = 0.035, *p* = 0.022 respectively, Wilcox). Phospho-tau (T181) is significantly upregulated in the hippocampus 30 min and 24 h post-5xCHI compared to sham-injured animals (*p* = 0.0058, *p* = 0.022 respectively, Wilcox) (mean ± SEM). **C**) Expression of the gene set “Kegg Alzheimer’s Disease” significantly increases after 1xCHI (*p* = 0.006, t-test with Bonferroni correction) (mean ± SEM). See individual gene changes in Fig. S10

Our motivation for conducting this study in 3xTg-AD mice was to identify relationships between immune signaling and molecular markers associated with AD-related pathology. To assess the relationship between our injury-elevated Group 1 immune signals and pathology-associated proteins, we conducted clustering and correlation analyses between Group 1 immune signals and pathological markers (total tau, phospho-tau (T181), Aβ 1–40, and Aβ 1–42). Few significant acute changes in Aβ or correlation with Group 1 proteins were observed in either region or sex (Figs. S6-7). However, in cortex and hippocampus in both sexes, the Group 1 immune markers clustered with phospho-tau with few exceptions (Fig. S4-5), suggesting a connection between cytokines and pathologically relevant tau phosphorylation [89]. Pearson’s correlation coefficients between phospho-tau T181 and each cortical immune marker within each injury group (Fig. 4A) revealed that the majority of immune signals in Group 1 were correlated (|R|>0.50) across multiple time points. These data thus suggest that injury drives acute cytokine and MAPK immune signaling associated with increased phosphorylated tau in this model.

Interestingly, we observed these pronounced correlations at multiple time points in the frontal cortex despite only finding group-wise elevation of phospho-tau at 24 h post-3xCHI in female 3xTg-AD frontal cortices (Fig. S8) and no group-wise changes in males. However, total tau and phospho-tau (T181) were elevated at multiple time points after 3x and 5xCHI in the female 3xTg-AD hippocampus (Fig. 4B, Fig. S8-9). These data thus indicate that our CHI model induces acute pathological changes in a region-dependent manner, and that Group 1 immune signaling correlates with phosphorylated tau even in the absence of group changes in total or phosphorylated tau in this model.

Having found group-wise tau differences in 3xTg-AD females, we asked if we could also detect an AD pathology transcriptional signature after rmTBI in females. To do so, we conducted bulk RNA sequencing on a subset of 40 3xTg-AD females (somatomotor cortex consisting of cortical tissue collected at a range of approximately bregma ± 1 mm, *n* = 3–4 per group, Table S2), then conducted gene set variation analysis (GSVA) using the Alzheimer’s disease-related gene set from the Molecular Signatures Database (MSigDB) C2 curated gene sets collection. This gene set was significantly enriched after a single injury and remained enriched with subsequent injuries (Fig. 4C, Fig. S10). Thus, our protein and transcriptional data together support pathological effects of rmTBI in this model, which are correlated with neuroimmune markers.

### Cytokines and MAPK phospho-proteins elevated after injury co-label with neurons

**Fig. 5:**
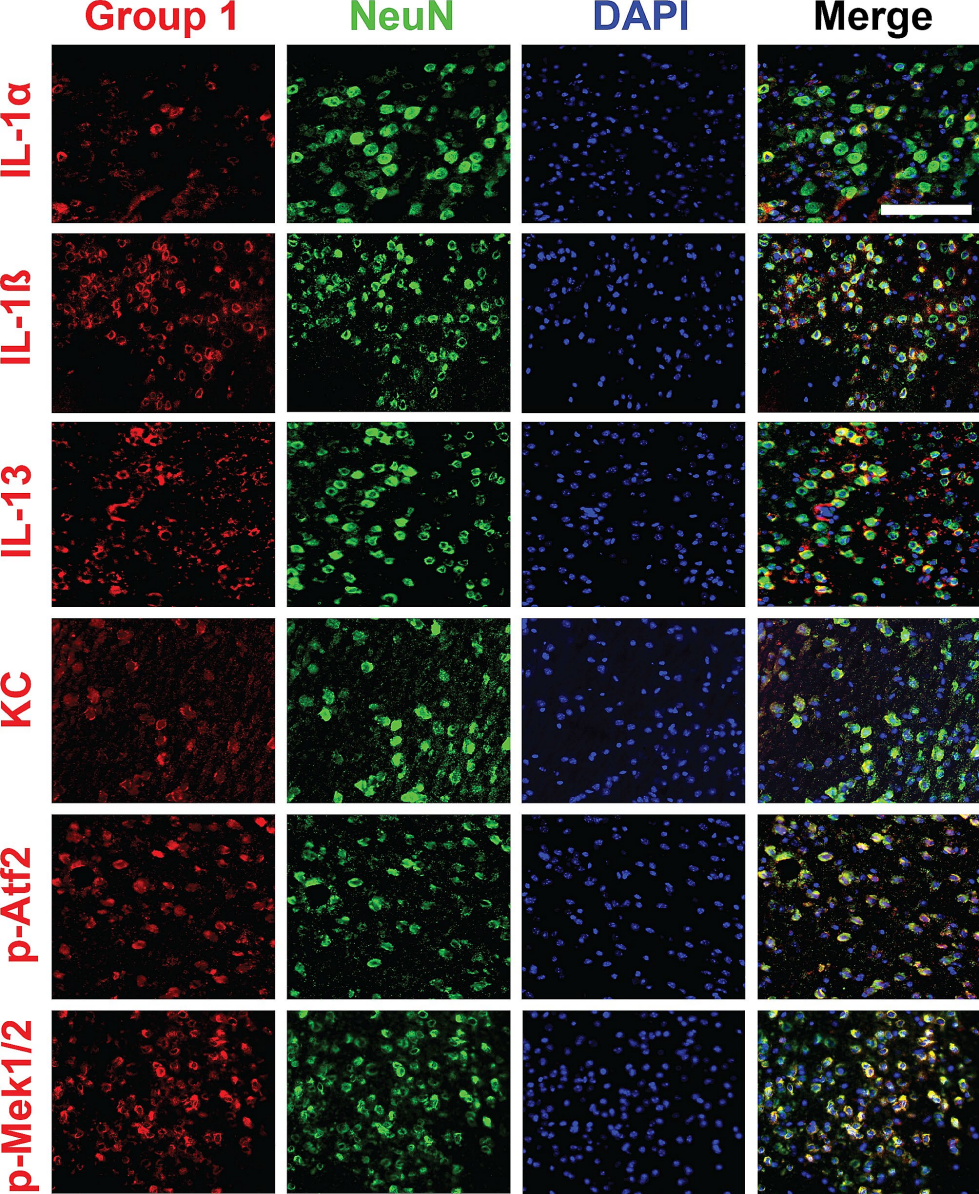
Group 1 cytokines and phospho-proteins co-label with NeuN in 3xTg-AD frontal cortex 24 h post-CHI. Group 1 cytokines (IL-1α, IL-1β, IL-13, and KC) and MAPK phospho-proteins (phospho-Atf2 and phospho-Mek1/2) (red) co-stained with NeuN (green) and DAPI (blue) show neuronal co-labeling in the 3xTg-AD frontal cortex 24 h post-1xCHI in female 3xTg-AD mice aged 2-4mo (scale bar: 500 μm, representative sections from *n* = 6 mice 24 h post-1xCHI)

Having found that Group 1 proteins correlated with phospho-tau, which is primarily a neuronal protein (Fig. 4A), we next asked if Group 1 signaling would be associated with neurons or other cell types. To this end, we conducted immunohistochemistry for various Group 1 proteins together with markers for neurons (NeuN) and astrocytes (GFAP) as a non-neuronal control at the 24 h time point in mice exposed to 1x or 3xCHI. We found that IL-1α, IL-1β, IL-13, phospho-Atf2, and phospho-Mek1/2 co-labeled with the neuronal marker NeuN in the cortex and hippocampus after injury, suggesting neuronal involvement in post-rmTBI immune signaling (Fig. 5, Fig. S11). Additionally, IL-1β, IL-13, KC, phospho-Atf2, and phospho-Mek1/2 did not co-labeled with the astrocyte marker GFAP in the cortex (Fig. S12) or hippocampus (Fig. S13). We further noted that many of these markers also co-labeled with NeuN in the frontal cortex of sham-injured animals (Fig. S14), which is consistent with our prior finding that neurons abundantly express cytokines and MAPK phospho-proteins under homeostatic conditions [91]. Collectively, these data indicate that neurons abundantly produce many of the cytokines and phospho-proteins that our quantitative assays (Fig. 3, S4, S5) show are affected by injury.

### Transcriptional and protein markers of astrocyte reactivity are increased after repetitive closed head injury

**Fig. 6:**
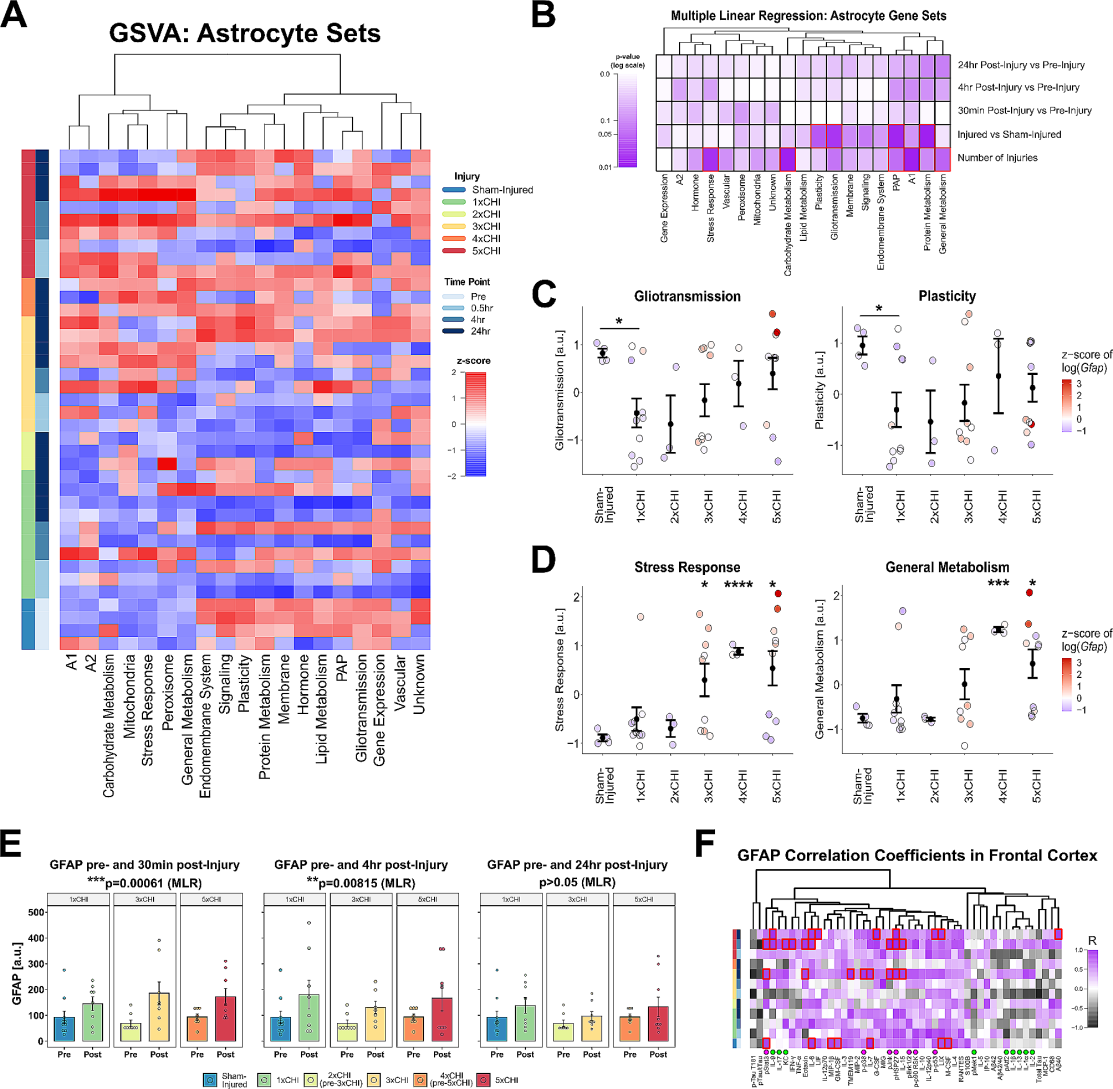
Cortical astrocytes become reactive following mild TBI. **A**) Gene set variation of custom-curated astrocyte gene sets (columns) across injury and time point groups (rows) reveals two distinct expression patterns: gradual increase by 3-5xCHI (left cluster) and rapid decrease after 1xCHI (right cluster). Gene sets are z-scored and clustered by Euclidean distance. **B**) Multiple linear regression models were calculated for each gene set using fixed effects of time point after most recent injury, binary injury status (injured, sham-injured), and number of injuries. The model shows that the effects of injury (both numeric and binary) outweigh time-dependent changes for most gene sets. Red boxes indicate statistical significance (*p* < 0.05). **C**) Astrocyte gene sets Gliotransmission and Plasticity are representative of right cluster, displaying a decrease after 1xCHI followed by return to sham-injured levels by 4-5xCHI (*p* = 0.013, *p* = 0.032, t-test with Bonferroni adjustment) (mean ± SEM). Data points are colored by the z-score of the log of astrocyte reactivity marker gene *Gfap*. **D**) Astrocyte gene sets Stress Response and General Metabolism are representative of left cluster, displaying a gradual increase after 3-5xCHI (Stress Response 3xCHI *p* = 0.038, 4xCHI *p* < 0.0001, 5xCHI *p* = 0.015; General Metabolism 4xCHI *p* = 0.00012, 5xCHI *p* = 0.020; t-test with Bonferroni adjustment) (mean ± SEM). Data points are colored by the z-score of the log of astrocyte reactivity marker gene *Gfap*, which is associated with high expression in either set. **E**) Glial fibrillary acidic protein (GFAP) is significantly upregulated 30 min and 4 h post-injury vs. pre-injury (*p* = 0.00061, *p* = 0.0082 respectively; multiple linear regression model, pre/post and number of injuries) (mean ± SEM). Note that sham-injured is equivalent to pre-1xCHI, 2xCHI at 24 h is equivalent to pre-3xCHI, and 4xCHI at 24 h is equivalent to pre-5xCHI in this representation. **F**) Heatmap of Pearson’s correlation coefficient of cortical GFAP versus each measured cortical protein (columns) within each injury group and time point (rows); red boxes indicate FDR-corrected *p* < 0.05. Panels **A**-**D** present transcriptional data from female somatomotor cortex. Panels **E**-**F** present protein data from female frontal cortex

Astrocyte reactivity is a central phenotype of brain injury. Several studies have reported elevated expression of the astrocyte reactivity marker GFAP that begins within hours and persists up to 1mo after TBI. GFAP is also being evaluated as a CSF biomarker for TBI diagnosis and prognosis [92–96]. We therefore hypothesized that astrocyte reactivity would be increased in 3xTg-AD mice exposed to successive mTBIs. To analyze possible changes in astrocyte phenotypes and function following injury, we conducted a gene set variation analysis (GSVA) [71, 84] of custom-annotated astrocyte gene sets that define astrocyte homeostatic and reactive functions (1,740 genes spanning 17 gene sets plus the A1 and A2 gene sets [97]) from our prior work [86] (Fig. 6A). To define the specific effects of injury and time point on astrocyte transcriptional profiles, we conducted multiple linear regression of the enrichment scores of all 17 astrocyte gene sets using number of injuries, time point after injury, and a binary injury variable (injured or sham-injured) as independent variables. This analysis revealed that the number of injuries had a greater effect on gene set enrichment than the post-injury time point (Fig. 6B). We therefore combined post-injury time points (30 min, 4 h, 24 h) to create a single group for each number of injuries 1-5xCHI, improving our ability to analyze differences despite our small sample sizes (*n* = 3–4 per time point).

Clustering of sample enrichment scores for the 17 astrocyte gene sets revealed two distinct patterns of expression after repeated injury (Fig. 6A). First, 12 astrocyte gene sets formed a cluster characterized by rapid decreases in enrichment following the first CHI, as represented by the Gliotransmission and Plasticity sets (Fig. 6C). These sets mostly showed recovery of expression to sham-injured levels by 24 h post-4/5xCHI. Many of these sets represent normal homeostatic astrocytic functions, such as gliotransmission, plasticity, gene expression, and the perisynaptic astrocyte process (PAP). A rapid decrease in expression of these sets following 1xCHI suggests astrocytes first respond to injury in this model by altering their homeostatic functions. The second response pattern, represented by Stress Response and General Metabolism (Fig. 6D), shows a gradual increase in expression after 3-5xCHI. Coloring data points according to *Gfap* revealed an association between astrocyte reactivity and elevated Stress Response and General Metabolism gene sets. Other gene sets exhibiting this trend of a gradual increase include the A1 and A2 phenotypes and Carbohydrate Metabolism, which are functions that are generally related to astrocyte reactivity [97, 98]. These data therefore suggest elevated astrocyte reactivity by 3-5xCHI, 72–120 h after the first daily injury.

We next quantified astrocyte reactivity markers glial fibrillary acidic protein (GFAP) and S100 calcium binding protein B (S100B) by ELISA in the frontal cortex and hippocampus of all female 3xTg-AD mice exposed to 1x, 3x, or 5x once daily CHIs. To assess GFAP in our injury model, we conducted a multiple linear regression analysis with dependent variables of time point following injury (pre-injury vs. 30 min, 4 h, or 24 h post-injury) and total number of injuries. Across female 3xTg-AD frontal cortex GFAP levels, we found that post-injury samples were significantly upregulated from pre-injury samples at both 30 min and 4 h (*p* = 0.00061, *p* = 0.0082 respectively; multiple linear regression model, pre/post and number of injuries), but not 24 h post-injury (Fig. 6E), and that the total number of injuries was not a significant predictor of GFAP levels. In a parallel analysis, multiple linear regression of cortical S100B in female 3xTg-AD mice similarly revealed statistically significant upregulation 30 min post-injury vs. pre-injury (*p* = 0.028) but not after 4–24 h (Fig. S15). Taken together, these data indicate a rapid, transient state of astrocyte reactivity following each repeated CHI that begins as early as 30 min, but returns to basal levels by 24 h. We also quantified microglial activation marker CD68 but found that changes were minimal and inconsistent between sex and region (Fig. S16).

Because astrocyte reactivity is a key component of the brain’s response to stress and injury, we next analyzed the relationships between GFAP protein levels, cytokine expression, and MAPK signaling activity. Among both female cortical and hippocampal samples, a group of MAPK phospho-proteins, termed “Group 2” (phospho-Stat3, phospho-Jnk, phospho-HSP27, phospho-p38, phospho-p90 RSK, phospho-Erk1/2, and phospho-p53), clustered tightly together across all injury groups and closely with GFAP (Fig. S4-5), indicating potential involvement of astrocyte reactivity in both brain regions. Therefore, we next computed Pearson’s correlation coefficients between GFAP and each measured immune marker within each injury and time group (Fig. 6F) and found broad positive correlations both before and after injury, emphasizing the involvement of astrocytes in brain immune signaling in this model.

### Co-expression network analysis reveals the effect of injury on pathology- and cell type-associated transcriptional modules

**Fig. 7:**
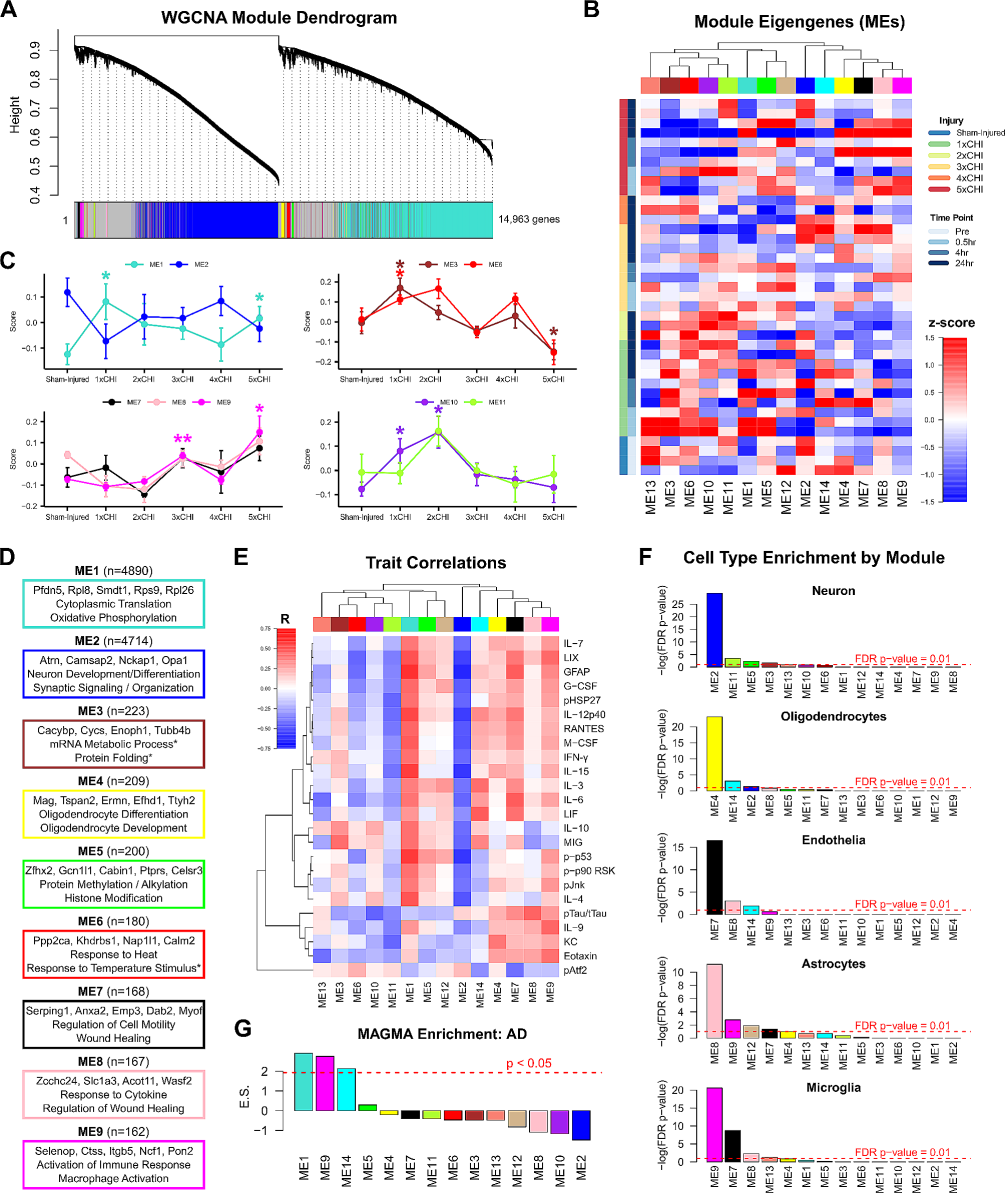
WGCNA of 3xTg-AD somatomotor cortex transcriptomes identifies modules of co-expressed genes associated with injury, GO biological processes, inflammatory protein expression, brain cell types, and AD genetic risk factors. **A**) WGCNA identified 14 modules of co-expressed genes. **B**) Expression scores for the 14 module eigengenes (MEs, the first principal component of each module) for each sample. MEs are clustered by Euclidean distance (columns) and expression scores are displayed by increasing injury number and time point from bottom to top of the heatmap (rows). **C**) Line plots show changing ME expression scores over increasing numbers of injuries. Time points for 30 min, 4 h, and 24 h after 1x, 3x, and 5xCHI are combined due to low sample sizes (*n* = 2–4). ME1 (turquoise) and ME2 (blue) show opposite behavior after 1xCHI, with ME1 significantly increasing (*p* = 0.047, permutation test) alongside trending decreases (*p* = 0.054) in ME2 after a single injury. ME 9 (magenta) shows significant increases after successive injuries that track closely with ME7 and 8 (black and pink) (*p* = 0.0015 after 3xCHI, *p* = 0.023 after 5xCHI; permutation tests compared to sham-injured; see Methods) (mean ± SEM). **D**) Gene ontology enrichment of biological processes was conducted for each module (Fisher’s exact test with FDR-corrected p-value < 0.05). ME3 and ME6 had fewer than two significant GO sets. Sets marked with asterisks had FDR-corrected *p* > 0.05 but uncorrected *p* < 0.0001. **E**) Protein expression data from the frontal cortex of the same 3xTg-AD animals were correlated against each ME. Selected proteins showed a correlation p-value of at least 0.01 against at least one ME. Red indicates a positive Pearson’s correlation coefficient (R) while blue indicates negative correlation. **F**) Cell type enrichment analysis showed significant enrichment of neuronal genes in ME2 (blue), oligodendrocyte genes in ME4 (yellow), endothelia genes in ME7 (black), astrocyte genes in ME8 (pink), and microglial genes in ME9 (magenta). **G**) MAGMA enrichment for GWAS AD genes showed significant over-representation within ME1 (turquoise), ME9 (magenta), and ME14 (cyan). The dotted red line indicates an enrichment z-score of 1.96, above which a module is considered significantly enriched (normal distribution, *p* < 0.05)

To holistically define the effects of repetitive mTBI in our model, we concluded the current study by conducting weighted gene co-expression network analysis (WGCNA) on the same subset of 40 female 3xTg-AD cortical samples used in Figs. 4C and 6 sampled from all injury groups and time points (*n* = 3–4 per group). One sample was removed from the analysis due to its outlier status and exclusion within the protein data (Mahalanobis multivariate outlier detector with α < 0.001, Methods). WGCNA identified 14 distinct modules of co-varying genes (Fig. 7A). A module eigengene (ME) was computed for each module as its first principal component, and expression scores of each ME were displayed for each sample across all injury and time point groups (Fig. 7B). Due to small sample sizes and relatively low variation within time points of each injury group (Fig. 7B), we combined post-injury time points (30 min, 4 h, 24 h) into one group per injury number and compared module expression across number of injuries. The two modules comprised of the largest number of genes, ME1 (turquoise) and ME2 (blue), rapidly changed after 1xCHI, with ME1 showing a significant increase (*p* = 0.047, permutation test compared to cham-injured; see **Methods**) and ME2 showing trending decreases (*p* = 0.054) post-injury. In contrast, ME9 (magenta) progressively increased with successive injuries (*p* = 0.0015 after 3xCHI, *p* = 0.023 after 5xCHI; permutation tests compared to sham-injured) (Fig. 7C) and tracked closely with ME7 and ME8 (black, pink) after 2-5xCHI. Moreover, ME3 (brown) and ME6 (red) were increased after 1xCHI (*p* = 0.030 brown, *p* = 0.043 red) then decreased compared to sham-injured after 5xCHI (*p* = 0.037 brown, *p* = 0.072 red), while ME10 (purple) and ME11 (green-yellow) peaked after 2xCHI (*p* = 0.029 purple, *p* = 0.11 green-yellow), then returned to sham-injured levels. These modules therefore define changes associated with rapid (ME1, ME2), transient (ME3, ME6, ME10, ME11) and gradual time scales (ME7, ME8, ME9).

To understand the biological relevance of each module, we used gene ontology (GO, via PANTHER) to identify top GO terms enriched in each module (Fig. 7D). The rapidly changing ME2 (blue) was enriched for genes related to nervous system development, suggesting a rapid downregulation of homeostatic neuronal functions. The gradually changing modules, ME7, ME8, and ME9 (black, pink, magenta), were enriched for GO terms associated with wound healing, cytokine response, gliogenesis, and regulation of the immune response, suggesting a multi-day response that involves immune processes and healing. Importantly, those modules that increase with injury were all correlated with immune proteins (inflammatory cytokines, MAPK activation, and GFAP) quantified in the frontal cortex of the same animals (ME1, ME7, ME9; “Trait Correlations” Fig. 7E), whereas ME2, which shows decreasing trends after injury and is associated with neuronal function, was inversely correlated with immune proteins (Fig. 7E).

We next conducted a cell-type enrichment analysis for genes with high specificity in neurons, oligodendrocytes, endothelia, astrocytes, and microglia derived from cell type specific mouse brain proteome and transcriptome studies [78, 79](Fig. 7F). We found that ME2 (blue), which decreases after a single injury and is inversely correlated with immune protein markers, was significantly enriched for neuronal genes. In contrast, ME7, ME8, and ME9 (black, pink, magenta), which show gradual increasing trends with successive injuries and (with the exception of ME8) correlate with immune protein markers, were enriched for endothelial, astrocyte, and microglial genes, respectively.

We concluded this unbiased analysis by asking if specific modules were associated with AD-related genes. To test this, we used a Multi-marker Analysis of GenoMic Annotation (MAGMA) analysis to determine if AD genes were significantly over-represented within specific gene modules. Indeed, AD genes were enriched in ME1 (i.e. *Pfdn5*, *Rps11*, *Rpl13a*, *Lamtor4*, *Rpl14*), which increases with a single injury and correlates significantly to several inflammatory proteins. AD genes were also enriched in ME9 (i.e. *Hepacam*, *Vav1*, *Hexb*, *Grn*, *Laptm5*), which increases gradually, correlates with inflammatory proteins, and is enriched for microglial genes (Fig. 7G). Taken together, these data suggest an early inflammatory response associated with AD genes (ME1) and a concomitant neuronal decrease in homeostatic functions (ME2) after a single injury in 3xTg-AD mice. Moreover, multiple injuries progressively increased gene expression profiles associated with wound healing and regulation of inflammation within endothelia, astrocytes, and microglia, emphasizing that repetitive injuries affect all cellular compartments.

## Discussion

In this study, we comprehensively profiled immune and pathological responses to rmTBI in the brains of 3xTg-AD mice using a combination of targeted protein panels and RNA sequencing. We hypothesized that repeated mTBI drives progressive changes in immune signaling together with markers of astrocyte reactivity, microglial activation, and changes in tau and Aβ. Because wild type mice do not develop plaques and tangles associated with AD or brain injury-induced pathology, we employed the triple transgenic model of AD (3xTg-AD) in the present study to evaluate relationships between injury, pathological hallmarks, and markers of immune response across multiple acute time points. To our knowledge, this study is the first to define the temporal evolution of immune and pathological responses to rmTBI at both the protein and transcriptional levels in a mouse model capable of displaying both Aβ plaques and neurofibrillary tangles.

While sex differences in pathology and cognitive outcomes after injury in 3xTg-AD mice have been extensively studied [87], few studies have evaluated sex differences in immune signaling. Indeed, to our knowledge, all published brain injury studies that make use of the 3xTg-AD model report only male mice or combined-sex experimental groups. Sexual dimorphism of brain immune signaling in both humans and other mouse models is well-documented, generally showing a stronger immune response in females [99]. Female 3x-Tg AD mice are reported to have increased Aβ pathology, possibly due to increased β-secretase processing [88, 100, 101] while male 3xTg-AD mice and have been reported to have increased systemic autoimmunity [102], emphasizing the need to evaluate the role of sex. Indeed, we found that 40 out of 46 measured proteins were significantly different between males and females in the frontal cortex, as well as 37 out of 46 in the hippocampus. Further, we determined that total tau, phospho-tau T181, Aβ40, and Aβ42 accounted for only half of the differentially expressed proteins. In particular, pathology-adjusted data left 18 significantly different proteins in the frontal cortex and 20 in the hippocampus. These findings emphasize the importance of evaluating the role of sex. Indeed, dichotomizing our protein data into male and female datasets revealed that females exhibited a much stronger response to injury (Fig. S4, Fig S5). For this reason, we focused our analysis on females.

A key finding of our work is that many of the quantified cytokines and phospho-protein signaling molecules co-labeled with NeuN. Neurons are known to express a plethora of cytokine receptors and undergo phospho-protein signaling cascades (i.e., MAPK and NF-κB, among others) in response to inflammation [103–108], but an increasing body of work suggests that neurons also participate in cytokine secretion in both homeostatic and pathological conditions [109–120]. Indeed, prior work from our own group has found increased MAPK and NF-κB signaling and expression of cytokines that co-label with neurons following rmTBI in male wild type mice [38]. Moreover, multiple studies have reported evidence of co-localization of immune signaling proteins and/or their transcripts with neurons in most of our “Group 1” proteins, which are elevated in the cortex in females following each CHI: IL-1α [109, 110], IL-1β [111, 112], IL-2 [113, 114], IL-9 [115], IL-13 [116–118], and KC. Our IHC in the current study revealed prominent NeuN co-labeling of four cytokines (IL-1α, IL-1β, IL-13, and KC) and both MAPK phospho-proteins (phospho-Atf2 and phospho-Mek1) within “Group 1,” which is consistent with prior literature. One caveat of our NeuN labeling in these drop-fixed brains is that there was reduced NeuN labeling in the nuclei of many cells. This may be an artifact of our drop fixation protocol [121], which we adopted to enable microdissection of the left hemisphere for protein and mRNA analysis. Although future IHC studies are warranted using a perfusion-fixation approach, our data together with prior literature therefore support a role for neurons in orchestrating an immune signaling response post-rmTBI. Our current findings are further supported by our prior work showing that numerous cytokines and phospho-proteins were neuron-derived in resting state brain via native-state proteomics [91], which will provide a quantitative approach to interrogate neuronal signaling in future rmTBI studies.

To our knowledge, our work is the first to conduct transcriptional profiling after successive mTBIs in 3xTg-AD mice. We used WGCNA to provide an unbiased summary of gene expression trends in the data, then assessed each identified gene module for enhanced expression of specific biological processes (gene ontology), cell type specific markers, and GWAS-identified markers of AD, as well as correlation with paired protein expression (Fig. 7). Indeed, we identified an early inflammatory response associated with AD-GWAS gene expression and protein translation after a single injury, paired with a concomitant decrease in neuronal-enriched functions associated with neuronal development and synaptic signaling. These changes after a single injury seemingly parallel our findings of increased 24 h cytokines after 1x or 3xCHI that are no longer elevated after 5xCHI at 24 h. Within days of repeated injury, there was a gradual increase of endothelia-, astrocyte-, and microglia-enriched gene modules associated with wound healing and regulation of inflammation, suggesting a possible “cumulative” response. We also found a significant enrichment of AD-GWAS risk genes within the microglia-enriched gene modules, suggesting microglial involvement in acute pathogenesis after injury. We note, however, that our CD68 ELISA data did not change with injury, and thus we did not focus our protein and histological analyses on microglia. Collectively, these data suggest that even a single mTBI elicits a change in genes associated with lost neuronal homeostasis, whereas repeated daily mTBIs yield changes that propagate into astrocytes, microglia, and endothelial cells. The appearance of both transient and accumulating aspects of repetitive closed head injuries may be essential to identifying the mechanisms that cause cumulative functional deficits.

Interestingly, our protein data showed that the astrocyte reactivity markers GFAP and S100B are transiently elevated at 30 min after each repetitive mTBI in the female 3xTg-AD frontal cortex. Although few studies have characterized such rapid changes in reactivity markers following closed-head injury, elevated GFAP and GFAP breakdown products are detectable in serum less than one hour after mild to severe TBI in mice [92–94]. This parallels findings in human patients, where serum GFAP levels rapidly increase within an hour then decline within 24–72 h following mild-to-severe injury [94–96, 122–124] as well as some mouse studies [18, 125], but is in contrast to several other studies in rodents showing sustained GFAP elevation up to two weeks post-injury [126–129]. Differences in rodent and injury models, sample type (serum, CSF, tissue lysate), and sex composition of experimental groups may account for some of these conflicting results. On the transcriptional level, we found that astrocyte-specific gene sets for stress response, general metabolism, and immune-related phenotypes A1 and A2 [97] showed gradual increases compared to sham-injured after 3-5xCHI, while homeostatic sets associated with gliotransmission and astrocyte plasticity showed decreased expression compared to sham-injured after just 1xCHI. These findings mirror previous studies which have identified elevated immune response genes in astrocytes 7 days post-severe TBI [130] and the lack of astrocyte-specific inflammatory gene changes 24 h after mild TBI [131]. Combined, our protein and transcriptional data indicate that astrocytes are responsive to (r)mTBI as early as 30 min after injury, while transcriptional changes in stress response pathways persist on the order of days.

The MAPK intracellular signaling pathway is a known mediator of astrocyte reactivity [132–134], including after a traumatic brain injury [48]. The clustering of female 3xTg-AD cortical GFAP and multiple MAPK phospho-proteins suggests that MAPK signaling may be responsible for the phenotypic transition to a reactive state in our model. Further, GFAP also correlates with a host of cytokines such as LIX, IL-7, IL-3, IL-12p40, RANTES, IL-15, M-CSF, G-CSF. These relationships generally exist independently of injury and suggest involvement within reactive astrocyte signaling, which peaks on a time scale of minutes-to-hours after each injury. Further studies should investigate the effect of MAPK signaling in astrocyte response to brain injury.

The current study has several limitations requiring future work. First, while the 3xTg-AD mouse model is becoming relatively popular for brain injury research [55–62], its predisposition towards spontaneous development of pathology due to the expression of three causal AD transgenes is not genetically representative of most human TBI patients. The use of human transgenes is necessary due to the lack of pathogenicity of native mouse tau and Aβ, while the use of mutations specifically associated with dementia is justified by the short life span of mice. Here, we mitigated the potential effects of pre-existing pathology on injury outcomes by using mice aged to 2–4 months, well before the reported build-up of Aβ and phosphorylated tau at 6 and 12 months, respectively [135, 136]. Second, while bulk tissue processing enabled appropriate sample sizes and comparison between 12 injury and time point groups, it also limited our ability to draw conclusions at the level of a single cell. Our bulk analysis highlights injury groups and time points of interest to be interrogated in future work for single cell analysis. Single cell profiling would identify how subtypes of neurons, glia, and other cells respond to successive mTBIs at a higher resolution than can be inferred by network analysis of bulk transcriptomic data. Specifically, single cell analysis may help clarify the extent to which neurons may be involved in immune signaling through cytokine expression and MAPK activation as seen in the injury-driven “Group 1”. Additionally, bulk and single cell RNAseq profiling are needed in both sexes in future work to determine which transcriptional changes are shared between or distinct to each sex. Third, our findings are purely correlative. While we have established evidence of a relationship between elevated neuroimmune signaling and outcome, future studies are required to establish whether this signaling indeed drives pathogenesis after rmTBI, or is merely correlated.

## Conclusions

In total, our data define an acute neuroimmune cascade of mild traumatic brain injury in 3xTg-AD mice, consisting of (i) an immediate decrease in neuronal homeostatic gene expression and an elevation of AD-associated genes after a single injury, (ii) elevation of a subset of cortical cytokines that correlated with phosphorylated tau and co-labeled with neurons in injured mice, and (iii) increased expression of non-neuronal genes suggesting glial reactivity within days of repeated injury. We provide resolution of these acute protein and transcriptional changes at a previously uncharacterized minutes-to-days time scale alongside the key experimental variables of sex, brain region, and number of injuries. Pronounced changes in neuronal gene expression that correlate with inflammatory protein expression and co-labeling of injury-elevated cytokines and MAPK phospho-proteins with neuronal marker NeuN suggest a key role for neurons in the modulation of neuroimmune activity following brain injury. Further, the association of phospho-Mek1 and phospho-Atf2, cytokines, and phospho-tau T181 after injury may represent a therapeutic avenue for regulating inflammation and acute pathological mechanisms. Future work should explore the causal functions of these key molecular signals.

### Data availability

The gene expression FASTQ files and count matrix that support the findings of this study have been deposited in the Gene Expression Omnibus (GEO) repository under series record GSE226838. Protein expression data and mouse metadata has been deposited in the Open Data Commons for Traumatic Brain Injury repository under DOI: 10.34945/F5ZK51. The ODC-TBI is a secure, cloud-based repository platform designed to share research data [137].

### Electronic supplementary material

Below is the link to the electronic supplementary material.

Supplementary Material 1

## Data Availability

The gene expression FASTQ files and count matrix that support the findings of this study have been deposited in the Gene Expression Omnibus (GEO) repository under series record GSE226838. Protein expression data and mouse metadata has been deposited in the Open Data Commons for Traumatic Brain Injury repository under DOI: 10.34945/F5ZK51. The ODC-TBI is a secure, cloud-based repository platform designed to share research data (123).

## Abbreviations

3xTg-AD: Triple transgenic Alzheimer’s disease mouse model
Aβ: Amyloid beta
ACURO: Animal Care and Use Review Office (U.S. Army MRDC)
AD: Alzheimer’s disease
APP: Amyloid precursor protein
ARRIVE: Animal Research: Reporting of In Vivo Experiments
ATP: Adenosine triphosphate
BSA: Bovine serum albumin
CD68: Cluster of differentiation 68
CHI: Closed-head injury
CRAN: Comprehensive R Archive Network
DAPI: 4′,6-diamidino-2-phenylindole
ELISA: Enzyme-linked immunosorbent assay
FDR: False discovery rate
G-CSF: Granulocyte colony-stimulating factor
GEO: Gene expression omnibus
GFAP: Glial fibrillary acidic protein
GM-CSF: Granulocyte macrophage colony-stimulating factor
GSVA: Gene set variation analysis
GWAS: Genome-wide association study
IACUC: Institutional Animal Care and Use Committee
Iba1: Ionized calcium binding adaptor molecule 1
IFN-γ: Interferon gamma
IHC: Immunohistochemistry
IL: Interleukin
IP-10: Interferon gamma-induced protein 10
KC: Keratinocyte chemoattractant
LIF: Leukemia inhibitory factor
LIX: Lipopolysaccharide-induced CXC chemokine
MAGMA: Multi-marker Analysis of GenoMic Annotation
MAPK: Mitogen-activated protein kinase
MAPT: Microtubule-associated protein tau
MCP-1: Monocyte chemoattractant protein 1
M-CSF: Macrophage colony-stimulating factor
MIG: Monokine induced by interferon gamma
MIP: Macrophage inflammatory protein
MRDC: Medical Research and Development Command (U.S. Army)
NCBI: National Center for Biotechnology Information
mTBI: Mild traumatic brain injury
PAP: Perisynaptic astrocyte processes
PCA: Principal component analysis
PD: Parkinson’s disease
PSEN1: Presenilin 1
pTau: Phospho-tau
RANTES: Regulated upon activation, normal T cell expressed and presumably secreted
rmTBI: Repetitive traumatic brain injury
RNA: Ribonucleic acid
S100B: S100 calcium binding protein B
TBI: Traumatic brain injury
TBST: Tris-buffered saline with tween
TMEM119: Transmembrane protein 119
TNF-α: Tumor necrosis factorα
tTau: Total tau
WGCNA: Weighted gene co-expression network analysis
