# Supplementary material for: Profiling the neuroimmune cascade in 3xTg-AD mice exposed to successive mild traumatic brain injuries
Source: J Neuroinflammation. 2024 Jun 13;21:156. doi: 10.1186/s12974-024-03128-1 (PMC11177462; doi:10.1186/s12974-024-03128-1)
Supplement: Supplementary file 1 — Supplementary Material 1 [file 12974_2024_3128_MOESM1_ESM.pdf]

Profiling the neuroimmune cascade in 3xTg-AD mice exposed to successive mild traumatic brain injuries

Alyssa F. Pybus[1], Sara Bitarafan[2], Rowan O. Brothers[1], Lilly Rohrer[1], Arushi Khaitan[1], Felix Rivera Moctezuma[2], Kareena Udeshi[3], Brae Davies[3], Sydney Triplett[1], Martin N. Griffin[3], Eric Dammer[4], Srikant Rangaraju[5], Erin M. Buckley[1,6,7,8,¥], Levi B. Wood[1,2,8,¥, *]

[1]Wallace H. Coulter Department of Biomedical Engineering, Georgia Institute of Technology and Emory University, Atlanta, GA, USA
[2]George W. Woodruff School of Mechanical Engineering, Georgia Institute of Technology, Atlanta, GA, USA
[3]School of Biological Sciences, Georgia Institute of Technology, Atlanta, GA, USA
[4]Center for Neurodegenerative Diseases, School of Medicine, Emory University, Atlanta, GA, USA
[5]Department of Neurology, School of Medicine, Emory University and School of Medicine, Yale University, New Haven, CT, USA
[6]Department of Pediatrics, School of Medicine, Emory University, Atlanta, GA, USA
[7]Children's Healthcare of Atlanta, Atlanta, GA, USA
[8]Parker H. Petit Institute for Bioengineering and Bioscience, Georgia Institute of Technology, Atlanta, GA, USA

[¥]*Equally contributing corresponding authors*
**To whom correspondence should be addressed*

**Erin M. Buckley**, PhD[¥, *]	**Levi B. Wood**, PhD[¥, *]
Associate Professor<br>Wallace H. Coulter Department of Biomedical Engineering, Georgia Institute of Technology and Emory University; Department of Pediatrics, School of Medicine, Emory University<br>1760 Haygood Dr NE<br>Atlanta, GA 30322, USA<br>E-mail: erin.buckley@emory.edu	Associate Professor<br>George W. Woodruff School of Mechanical Engineering, Wallace H. Coulter Department of Biomedical Engineering, and Parker H. Petit Institute for Bioengineering and Bioscience, Georgia Institute of Technology<br>315 Ferst Dr, Rm 3311<br>Atlanta, GA 30332, USA<br>E-mail: levi.wood@me.gatech.edu

Supplementary Table S1. Research Resource Identifiers.

Resource	Catalog Number	RRID
3xTg-AD on C57BL/6J	Jackson Lab 033930	IMSR_JAX:033930
Anti-GFAP	Diagnostic Biosystems Mob064	Not Listed
Anti-NeuN	Abcam ab104224	AB_10711040
Anti-IL-1α	Thermo Fisher PA5-89037	AB_2805312
Anti-IL-1β	Abcam ab283818	Not Listed
Anti-IL-13	Abcam ab106732	AB_10867235
Anti-KC	Thermo Fisher PA5-86508	AB_2803283
Anti-phospho-Atf2 T71	Thermo Fisher PA5-97332	AB_2809134
Anti-phospho-Mek1/2 S217/S221	Thermo Fisher PA5-105777	AB_2817176
Anti-phospho-tau S262/T263	Abcam ab92627	AB_10563129

Supplementary Table S2. Sample sizes of each experimental group. Tables are separated by Female (top) and Male (bottom) samples by injury group (1x,3x,5xCHI) and time point (pre-injury, 30min, 4hr, 24hr). Sample size is indicated in n column. In total, 96 females and 83 males were used in the protein analysis and 39 females in RNAseq analysis. Two female and three male samples were excluded from the original 98 females and 86 males after Mahalanobis outlier detection (see **Methods**). *Pre-Injury 3xCHI is equivalent to 24hr post-2xCHI. **Pre-Injury 5xCHI is equivalent to 24hr post-4xCHI.

Protein Data

Sex	Injury	Time Point	Hr Since Start	n
Females	1xCHI	Sham-Injured	0	10
		30min	0.5	8
		4hr	4	8
		24hr	24	8
	3xCHI	Pre-Injury*	48	8
		30min	48.5	8
		4hr	52	6
		24hr	72	8
	5xCHI	Pre-Injury**	96	8
		30min	96.5	8
		4hr	100	7
		24hr	120	9
			Female Total:	**96**
Males	1xCHI	Sham-Injured	0	13
		30min	0.5	7
		4hr	4	6
		24hr	24	6
	3xCHI	Pre-Injury*	48	7
		30min	48.5	6
		4hr	52	7
		24hr	72	7
	5xCHI	Pre-Injury**	96	6
		30min	96.5	6
		4hr	100	6
		24hr	120	6
			Male Total:	**83**

RNAseq Data

Sex	Injury	Time Point	Hr Since Start	n
Females	1xCHI	Sham-Injured	0	4
		30min	0.5	3
		4hr	4	3
		24hr	24	4
	3xCHI	Pre-Injury*	48	3
		30min	48.5	3
		4hr	52	2
		24hr	72	4
	5xCHI	Pre-Injury**	96	3
		30min	96.5	3
		4hr	100	3
		24hr	120	4
			Female Total:	**39**

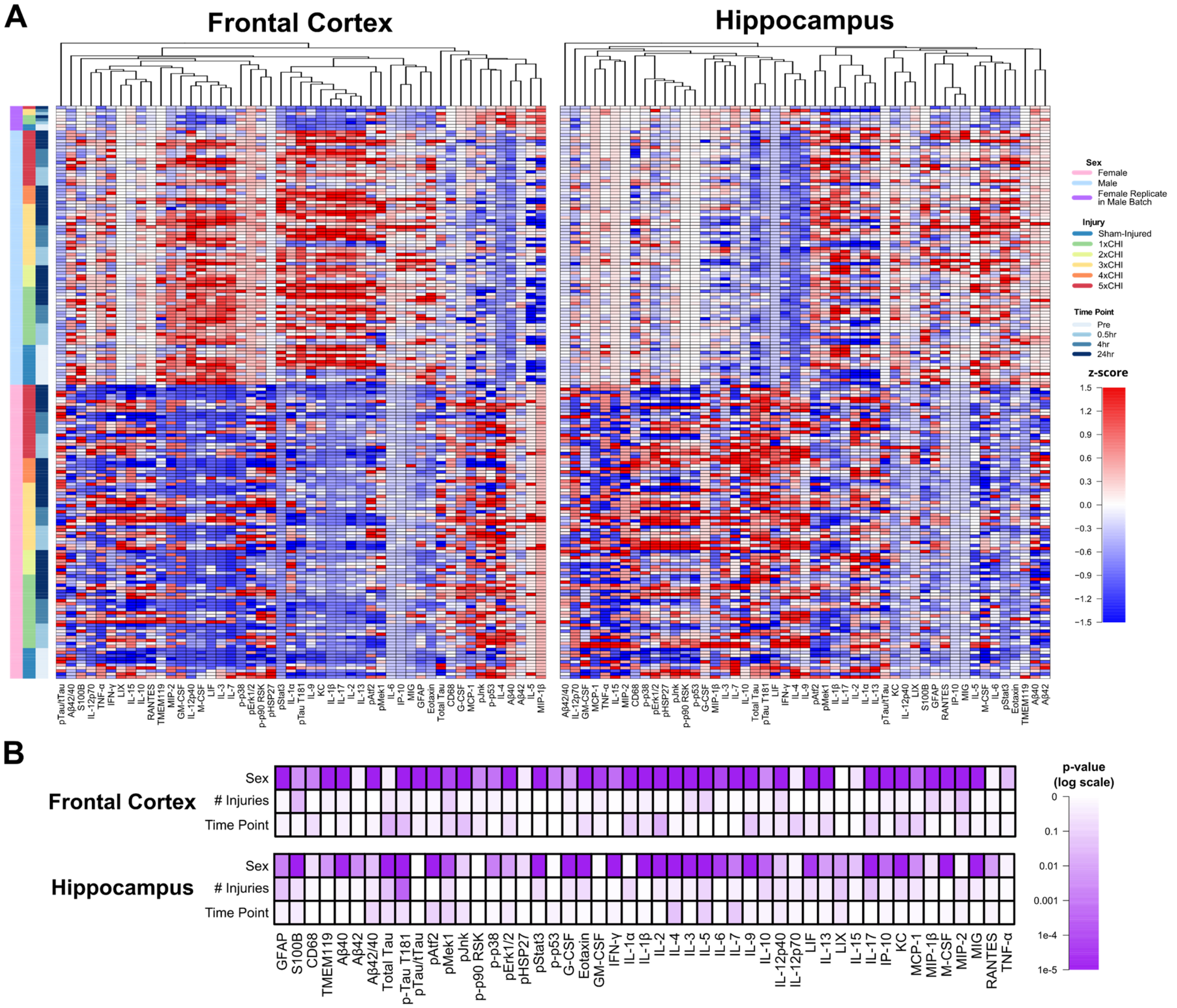

Supplementary Figure S1. Pronounced sexual dimorphism of immune signaling, glial markers, and molecular markers of pathology in 3xTg-AD mice: all-samples heatmap and multiple linear regression model. A) 30 cytokines, 6 MAPK phosphoproteins, amyloid beta 40 and 42, total and phospho-tau, GFAP, S100B, CD68, and TMEM119 were measured by multiplex and traditional ELISA in both frontal cortex (left) and hippocampus (right) tissue of 96 female (pink) and 83 male (blue) 3xTg-AD mice aged 2-4mo. Eight female samples were run as replicates within male batch assays (purple) to allow transformation of male data to female space by linear regression. S100B, CD68, and TMEM119 were run with all samples at the same time while all other assays were run in separate batches for male and female samples. Each row represents data from an individual mouse z-scored along each column. Columns are clustered by Euclidean distance via unweighted pair group method with arithmetic mean. **B**) Multiple linear regression models were calculated for each measured protein using fixed effects of sex, number of injuries, and time point after most recent injury. The model shows that the effect of sex strongly outweighs injury-dependent changes for most proteins from both regions.

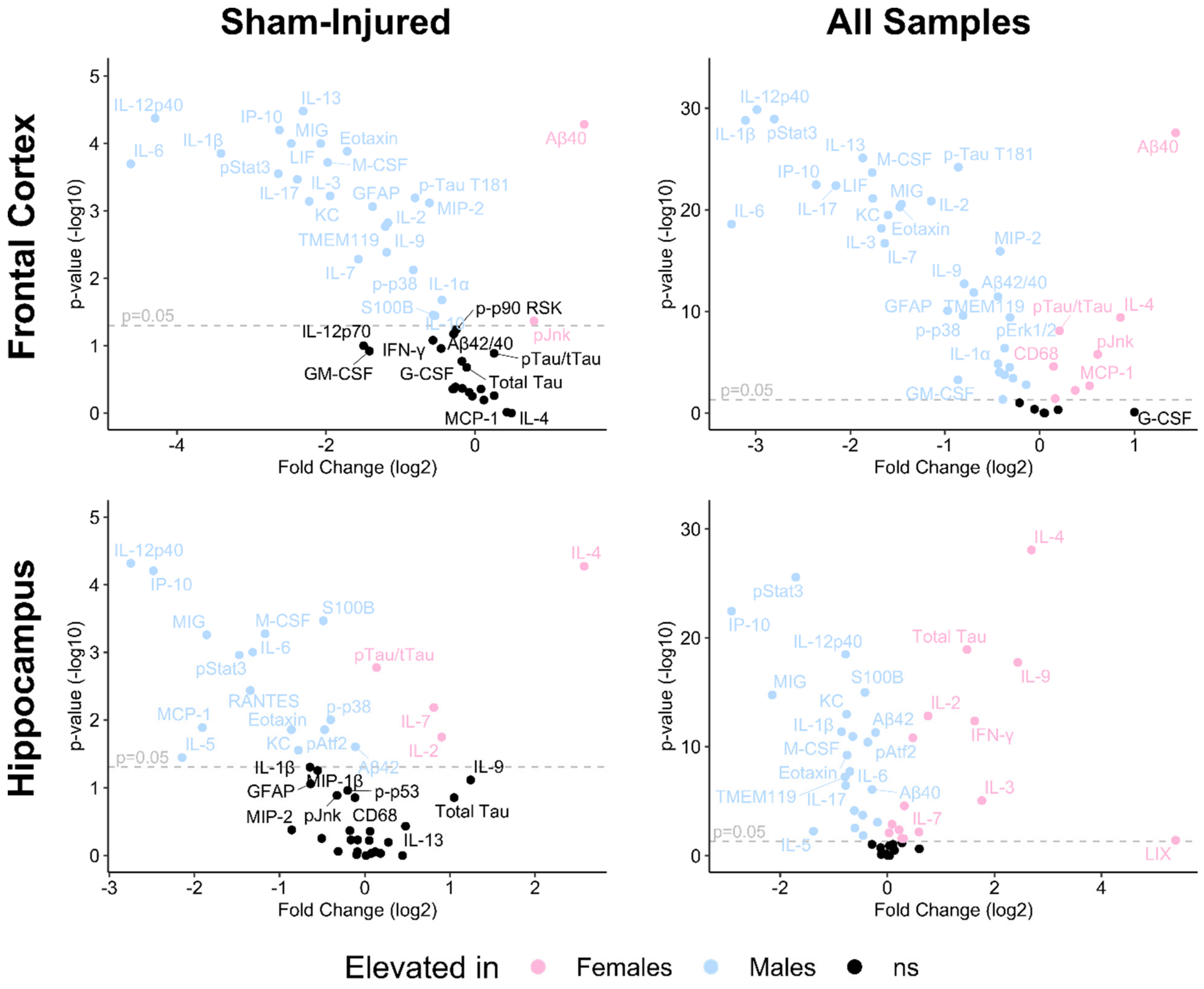

Supplementary Figure S2. Pronounced sexual dimorphism of immune signaling, glial markers, and molecular markers of pathology in 3xTg-AD mice: volcano plots. Volcano plots of male vs female comparative analysis show Wilcoxon rank-sum p-values (vertical axis, -log10 scale) and fold change (horizontal axis, log2 scale) of each measured protein. Proteins are considered significantly elevated in either male (blue) or female (pink) samples if $p<0.05$ by Wilcoxon rank-sum test. All significantly different proteins within sham-injured samples were also significant within all samples in the same direction. The all-samples analysis revealed a greater proportion of significantly different proteins by sex.

Supplementary Table S3. Sex differences by protein within each brain region among sham-injured and all samples. Proteins are considered significantly elevated in either male (blue) or female (pink) samples if p<0.05 by Wilcoxon rank-sum test. All significantly different proteins within sham-injured samples were also significant within all samples in the same direction. ns = not significant, *p<0.05, **p<0.01, ***p<0.001, ****p<0.0001. The all-samples analysis revealed a greater proportion of significantly different proteins by sex.

**Protein**	**Frontal Cortex Sham-injured**	**Frontal Cortex All**	**Hippocampus Sham-injured**	**Hippocampus All**
GFAP	Males ***	Males ****	ns	Males *
S100B	Males *	Males ***	Males ***	Males ****
CD68	ns	Females ****	ns	ns
TMEM119	Males **	Males ****	ns	Males ****
Aβ40	Females ****	Females ****	ns	Males ****
Aβ42	ns	Females *	Males *	Males ****
Total Tau	ns	ns	ns	Females ****
p-Tau	Males ***	Males ****	ns	Females ****
pAtf2	ns	Males ****	Males *	Males ****
pMek1	ns	Males ****	ns	Males ***
pJnk	Females *	Females ****	ns	ns
p-p90 RSK	ns	Males **	N/A	N/A
p-p38	Males **	Males ****	Males *	Males ***
pErk1/2	ns	Males ****	ns	Females **
pHSP27	ns	ns	ns	ns
pStat3	Males ***	Males ****	Males **	Males ****
p-p53	ns	Females **	ns	ns
G-CSF	ns	ns	Females **	Females ****
Eotaxin	Males ***	Males ****	Males *	Males ****
GM-CSF	ns	Males ***	ns	Females **
IFN-γ	ns	Males ***	ns	Females ****
IL-1α	Males *	Males ****	ns	ns
IL-1β	Males ***	Males ****	ns	Males ****
IL-2	Males **	Males ****	Females *	Females ****
IL-4	ns	Females ****	Females ****	Females ****
IL-3	Males ***	Males ****	Females *	Females ****
IL-5	Females *	Females ****	Males *	Males **
IL-6	Males ***	Males ****	Males **	Males ****
IL-7	Males **	Males ****	Females **	Females ****
IL-9	Males **	Males ****	ns	Females ****
IL-10	Males *	Males ****	ns	Females **
IL-12p40	Males ****	Males ****	Males ****	Males ****
IL-12p70	ns	Ns	ns	ns
LIF	Males ***	Males ****	ns	ns
IL-13	Males ****	Males ****	ns	Females *
LIX	N/A	N/A	ns	Females *
IL-15	ns	ns	ns	ns
IL-17	Males ***	Males ****	ns	Males ****
IP-10	Males ****	Males ****	Males ****	Males ****
KC	Males ***	Males ****	Males *	Males ****
MCP-1	ns	Females **	Males *	Males ****
MIP-1β	Females **	Females ****	ns	Females *
M-CSF	Males ***	Males ****	Males ***	Males ****
MIP-2	Males ***	Males ****	ns	Females **
MIG	Males ***	Males ****	Males ***	Males ****
RANTES	ns	ns	Males **	Males **
TNF-α	ns	Males *	ns	ns

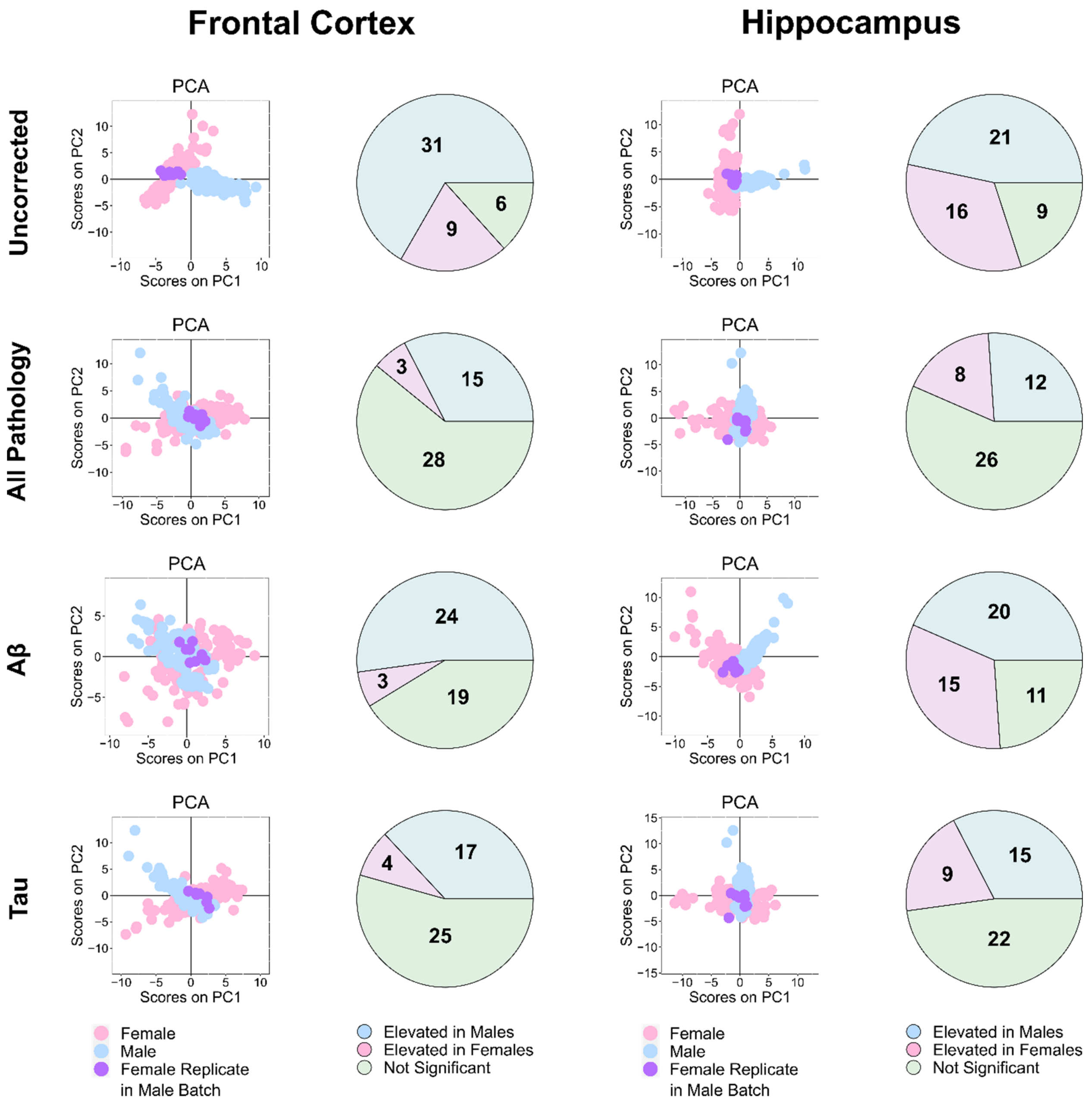

Supplementary Figure S3. Sex differences within pathology-adjusted data. Adjusted data is defined as the residual of a linear regression of Aβ markers (Aβ40 + Aβ42), tau markers (total tau + phospho-tau T181), or all pathological markers (Aβ and tau combined) against all combined protein data. From top to bottom, the graphic shows uncorrected data, all pathology-, Aβ-, and tau-adjusted data for both the frontal cortex (left) and hippocampus (right). A principal component analysis (PCA) scores plot of the first two PCs is shown to illustrate changes in variance between the samples after adjustment. The pie charts are annotated with the number of differentially expressed proteins which are significantly upregulated in male (blue) and female (pink) samples, or not significantly different (green). Proteins are considered significantly different if $p<0.05$ by Wilcox Rank Sum test.

Supplementary Table S4. Sex differences by protein within each brain region before and after adjustment for pathological markers. Proteins are considered significantly elevated in either male (blue) or female (pink) samples if p<0.05 by Wilcoxon rank-sum test. Adjusted data is defined as the residual of a linear regression of Aβ markers (Aβ40 + Aβ42), tau markers (total tau + phospho-tau T181), or all pathological markers (Aβ and tau combined) against all combined protein data. Phospho-p90 RSK and LIX were measured below the detection limit among all eight female replicate samples within the male batch hippocampus and frontal cortex, respectively, disallowing conversion to female space.

	Frontal Cortex				Hippocampus			
Protein	Original	All	Aβ	Tau	Original	All	Aβ	Tau
GFAP	M	ns	ns	M	M	ns	ns	ns
S100B	M	ns	ns	ns	M	M	M	M
CD68	F	F	F	F	ns	ns	ns	ns
TMEM119	M	M	ns	M	M	ns	M	ns
Aβ40	F	F	M	F	M	M	M	M
Aβ42	F	M	M	M	M	F	ns	M
Total Tau	ns	ns	ns	M	F	ns	F	M
Phospho-Tau	M	M	M	ns	F	ns	F	M
pAtf2	M	ns	M	ns	M	M	M	M
pMek1	M	ns	ns	ns	M	M	M	M
pJnk	F	ns	ns	ns	ns	ns	ns	ns
p-p90 RSK	M	ns	ns	ns	N/A	N/A	N/A	N/A
p-p38	M	M	M	M	M	ns	M	M
pErk1/2	M	M	M	M	F	ns	F	ns
pHSP27	ns	ns	ns	ns	ns	ns	F	ns
pStat3	M	ns	M	ns	M	M	M	M
p-p53	F	ns	ns	ns	ns	ns	ns	ns
G-CSF	ns	ns	ns	ns	F	ns	F	ns
Eotaxin	M	ns	ns	ns	M	ns	M	ns
GM-CSF	M	M	M	M	F	M	M	F
IFN-γ	M	ns	M	ns	F	F	F	F
IL-1α	M	ns	ns	ns	ns	ns	ns	ns
IL-1β	M	ns	M	ns	M	M	M	M
IL-2	M	ns	M	ns	F	F	F	F
IL-4	F	ns	F	F	F	F	F	F
IL-3	M	M	M	M	F	F	F	F
IL-5	F	ns	ns	ns	M	M	M	M
IL-6	M	ns	ns	M	M	ns	M	ns
IL-7	M	M	M	M	F	ns	F	ns
IL-9	M	ns	M	ns	F	F	F	F
IL-10	M	M	M	M	F	ns	F	ns
IL-12p40	M	M	M	M	M	ns	M	ns
IL-12p70	ns	M	ns	M	ns	ns	ns	ns
LIF	M	M	M	M	ns	F	ns	F
IL-13	M	ns	M	ns	F	ns	F	ns
LIX	N/A	N/A	N/A	N/A	F	ns	F	ns
IL-15	ns	ns	ns	ns	ns	ns	ns	ns
IL-17	M	ns	M	ns	M	M	M	M
IP-10	M	M	ns	ns	M	ns	M	ns
KC	M	ns	M	ns	M	M	M	M
MCP-1	F	ns	ns	ns	M	M	M	M
MIP-1β	F	F	F	F	F	ns	ns	F
M-CSF	M	M	M	M	M	M	M	M
MIP-2	M	ns	M	M	F	F	F	F
MIG	M	ns	ns	M	M	ns	M	ns
RANTES	ns	M	M	ns	M	ns	M	ns
TNF-α	M	ns	M	ns	ns	ns	ns	ns

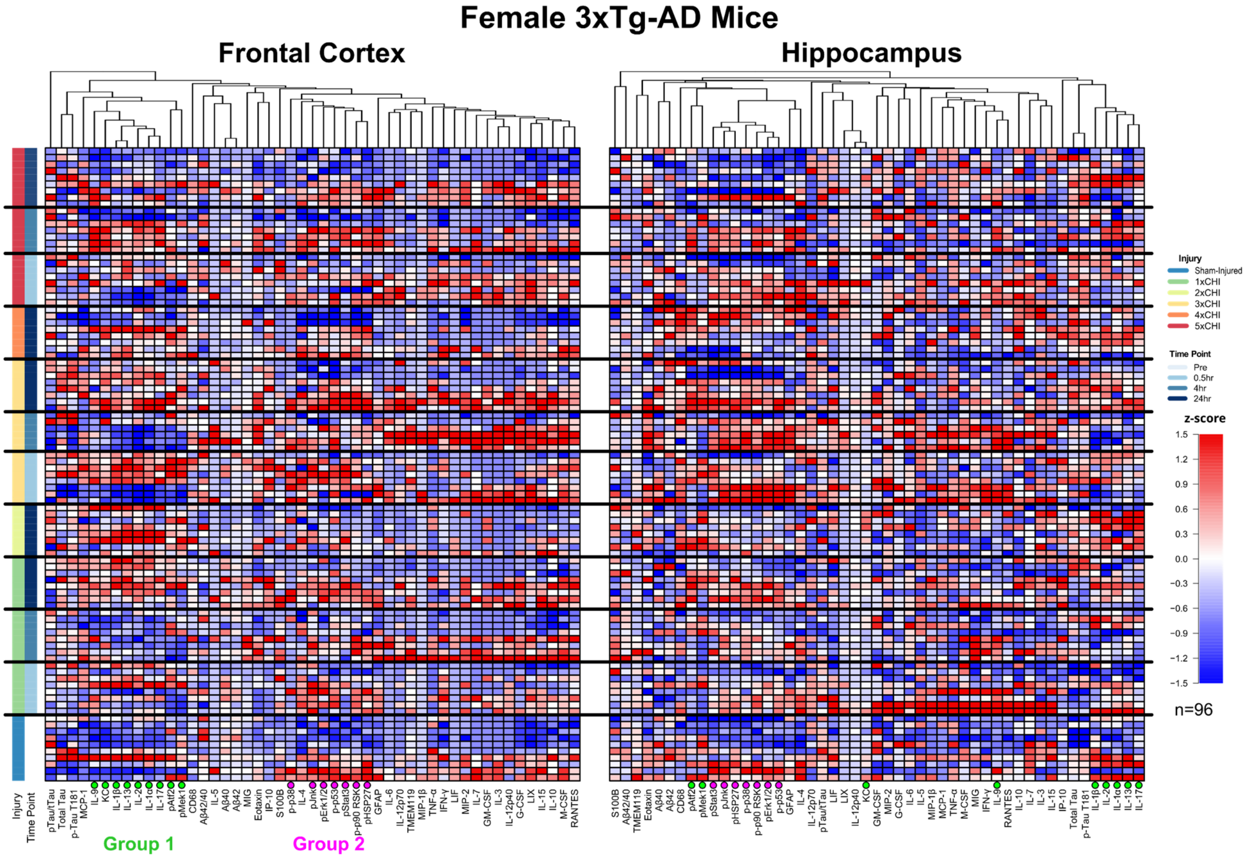

Supplementary Figure S4. Female protein data: Quantification of immune and pathological markers in female 3xTg-AD mice after Sham-Injury and the 30min, 4hr, & 24hr time points after 1x-5xCHI. 30 cytokines, 6 MAPK phospho-proteins, amyloid beta 40 and 42, total and phospho-tau, GFAP, S100B, CD68, and TMEM119 were measured by multiplex and traditional ELISA in both frontal cortex (left) and hippocampus (right) tissue of 96 female 3xTg-AD mice aged 2-4mo. Each row represents data from an individual mouse z-scored along each column. Columns are clustered by Euclidean distance via unweighted pair group method with arithmetic mean.

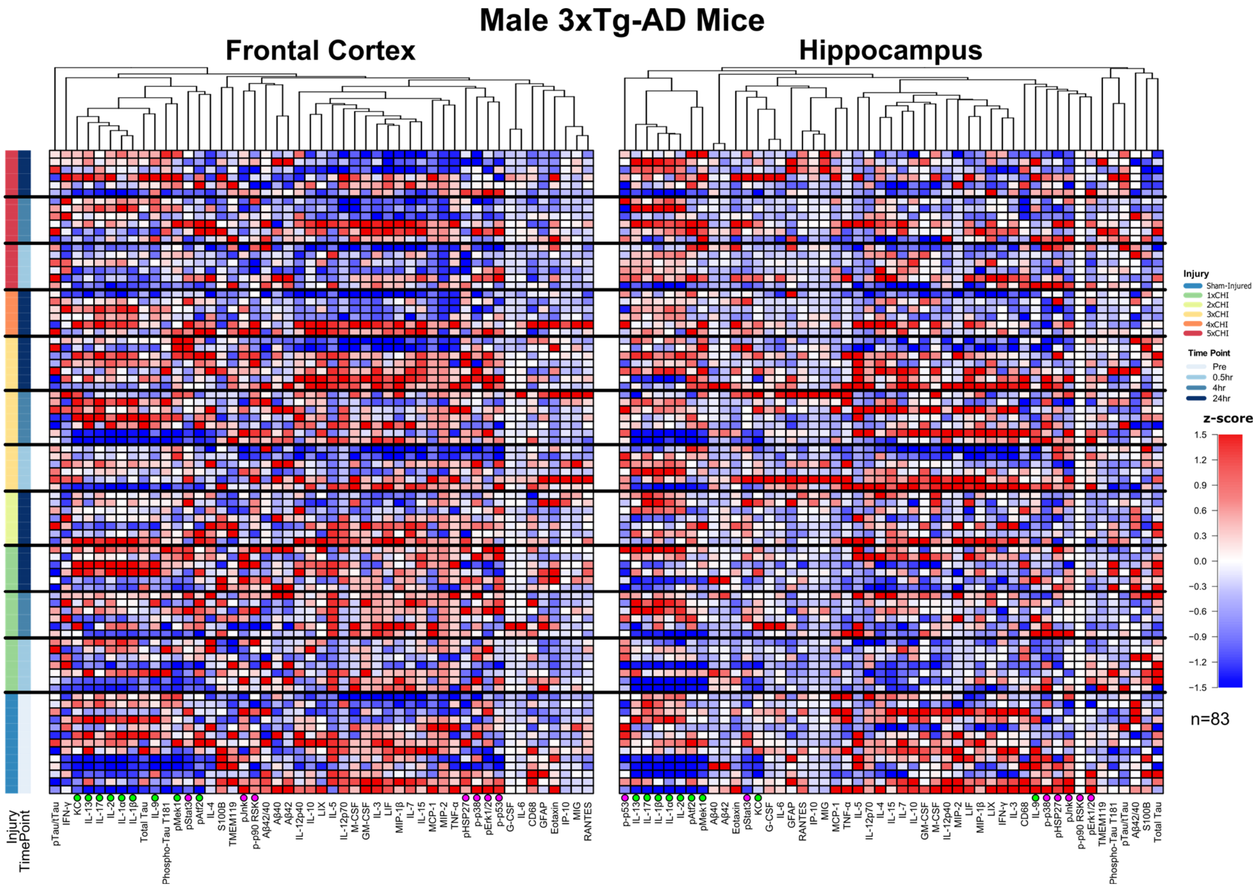

Supplementary Figure S5. Male protein data: Quantification of immune and pathological markers in male 3xTg-AD mice after Sham-Injury and the 30min, 4hr, & 24hr time points after 1x-5xCHI. 30 cytokines, 6 MAPK phospho-proteins, amyloid beta 40 and 42, total and phospho-tau, GFAP, S100B, CD68, and TMEM119 were measured by multiplex and traditional ELISA in both frontal cortex (left) and hippocampus (right) tissue of 83 male 3xTg-AD mice aged 2-4mo. Each row represents data from an individual mouse z-scored along each column. Columns are clustered by Euclidean distance via unweighted pair group method with arithmetic mean.

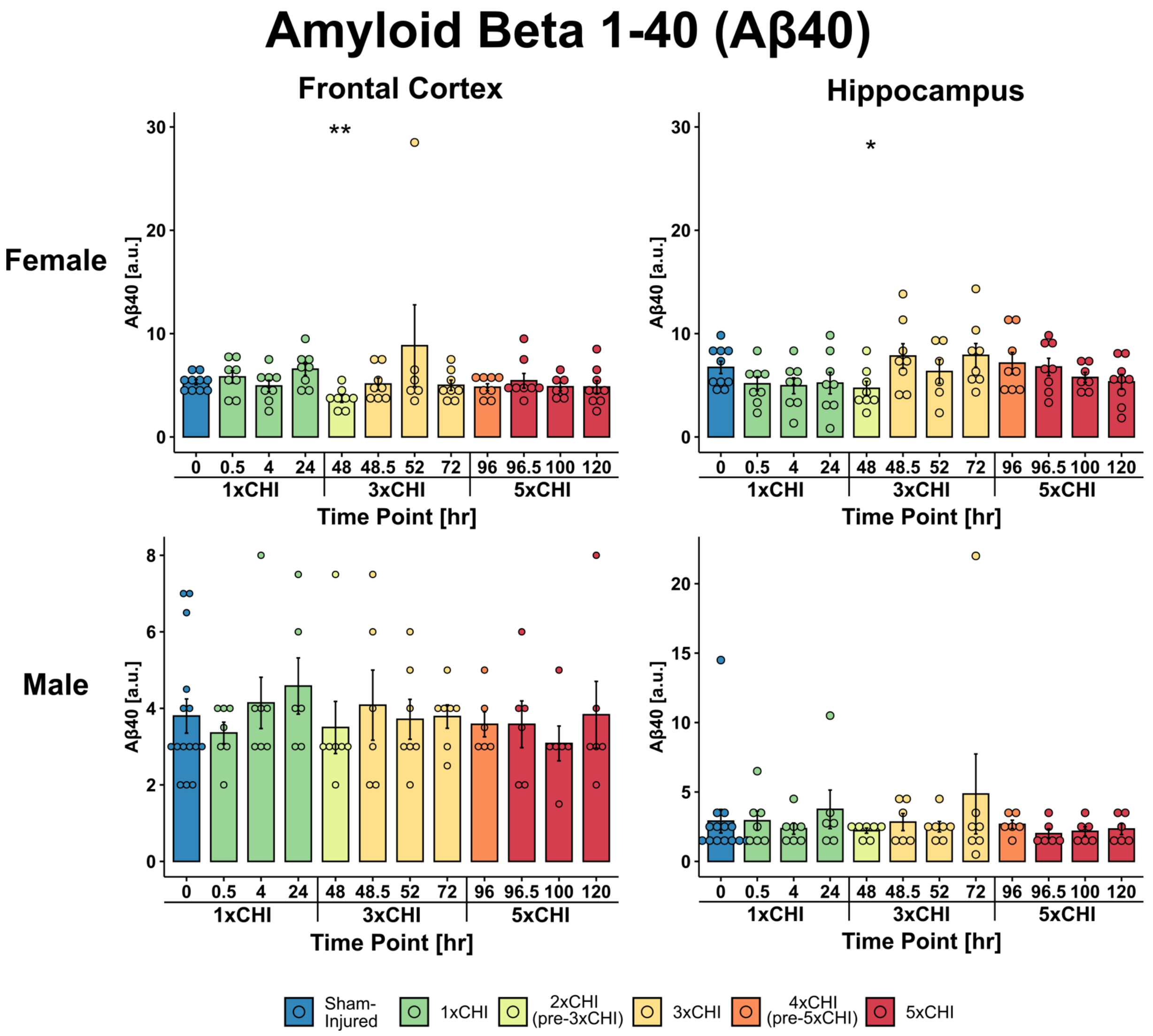

Supplementary Figure S6. Amyloid Beta 1-40 (Aβ40) by sex and brain region. Aβ40 was measured by Luminex ELISA (Millipore Sigma HNABTMAG-68K) in male and female frontal cortex and hippocampus tissue (*p<0.05, **p<0.01, Wilcox test vs sham-injured) (mean ± SEM). Male and female samples run separately.

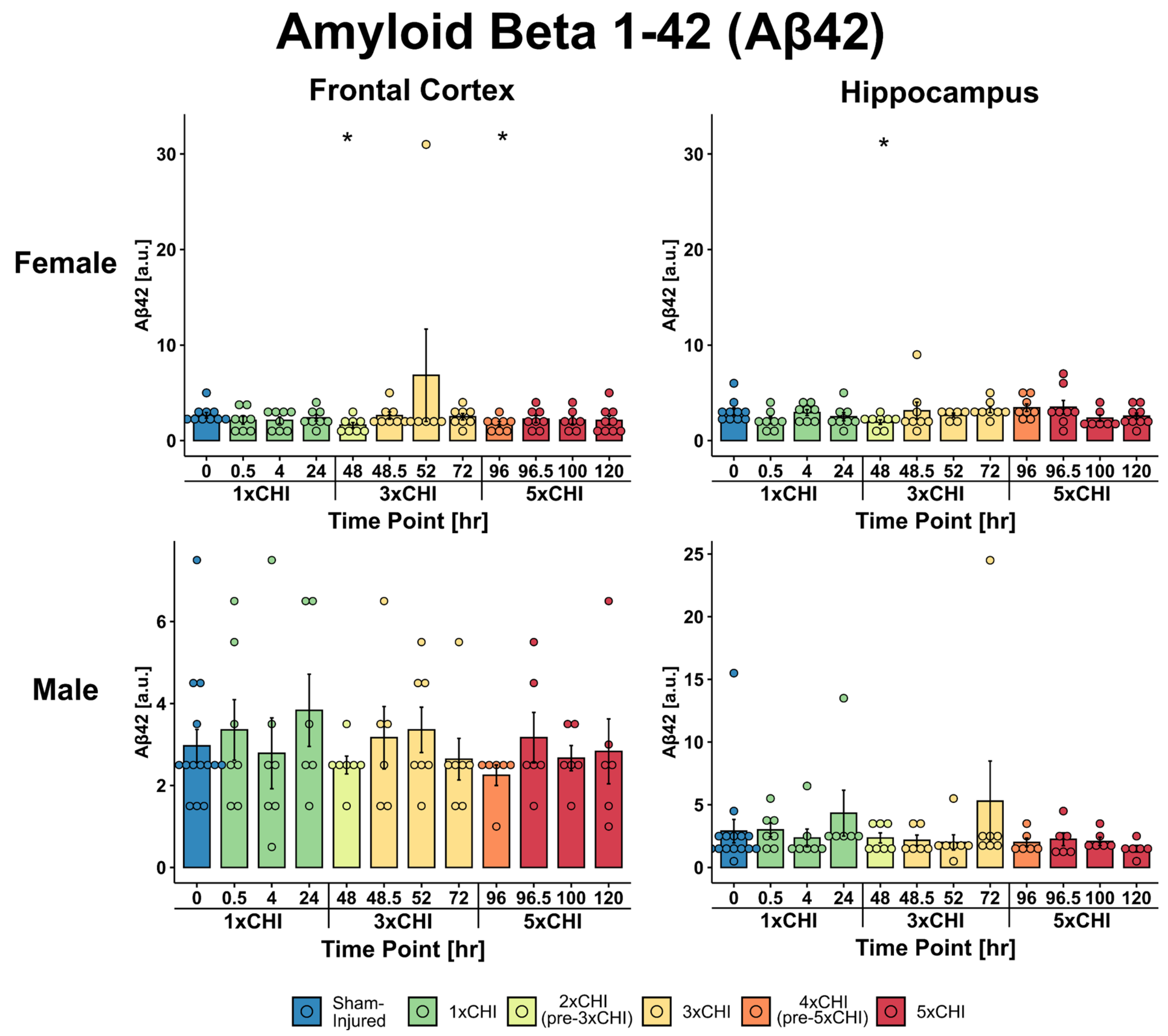

Supplementary Figure S7. Amyloid Beta 1-42 (Aβ42) by sex and brain region. Aβ42 was measured by Luminex ELISA (Millipore Sigma HNABTMAG-68K) in male and female frontal cortex and hippocampus tissue (*p<0.05, Wilcox test vs sham-injured) (mean ± SEM). Male and female samples run separately.

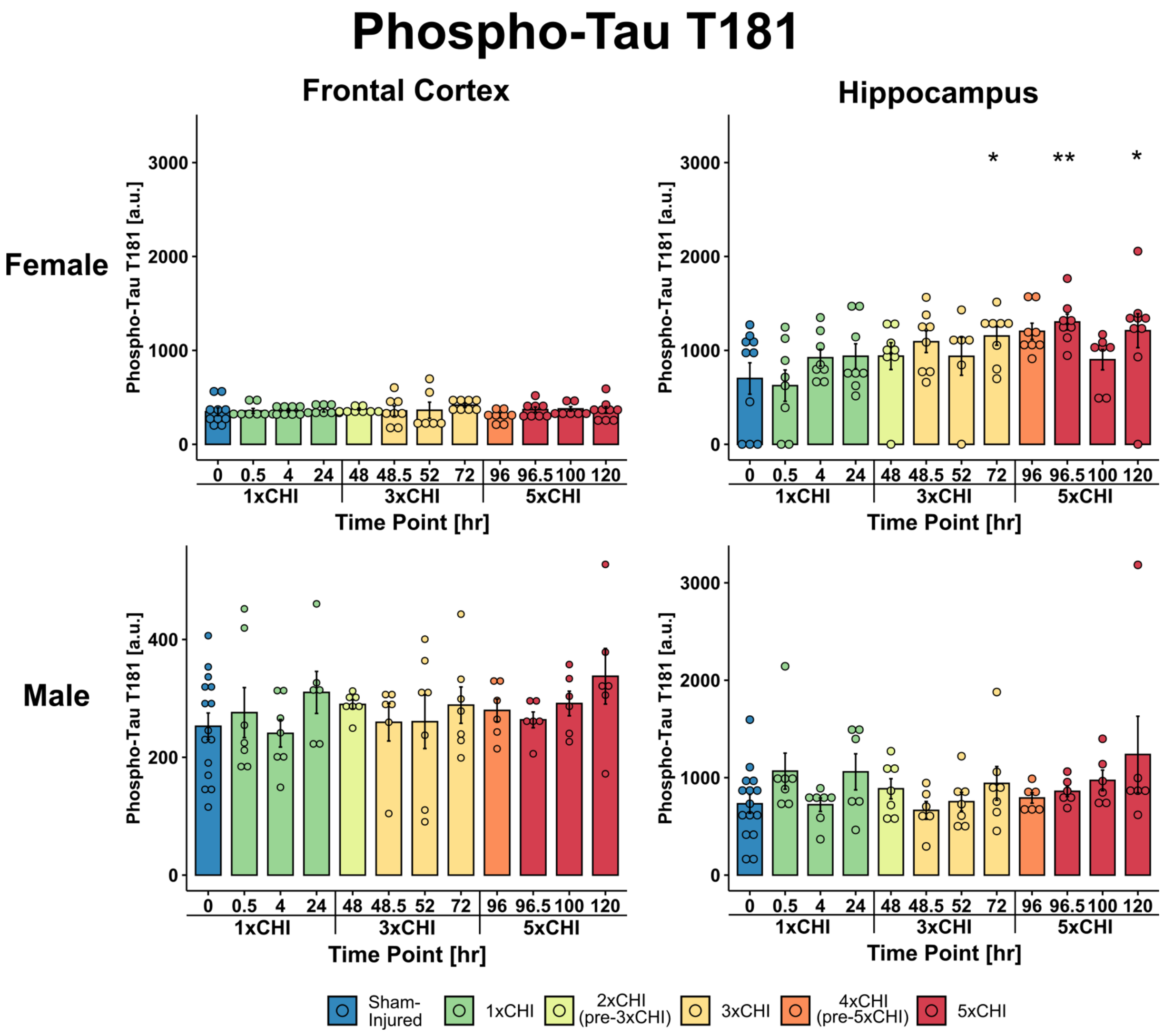

Supplementary Figure S8. Phospho-tau T181 by sex and brain region. Phospho-tau T181 was measured by Luminex ELISA (Millipore Sigma HNABTMAG-68K) in male and female frontal cortex and hippocampus tissue (*p<0.05, **p<0.01, Wilcox test vs sham-injured) (mean ± SEM). Male and female samples run separately.

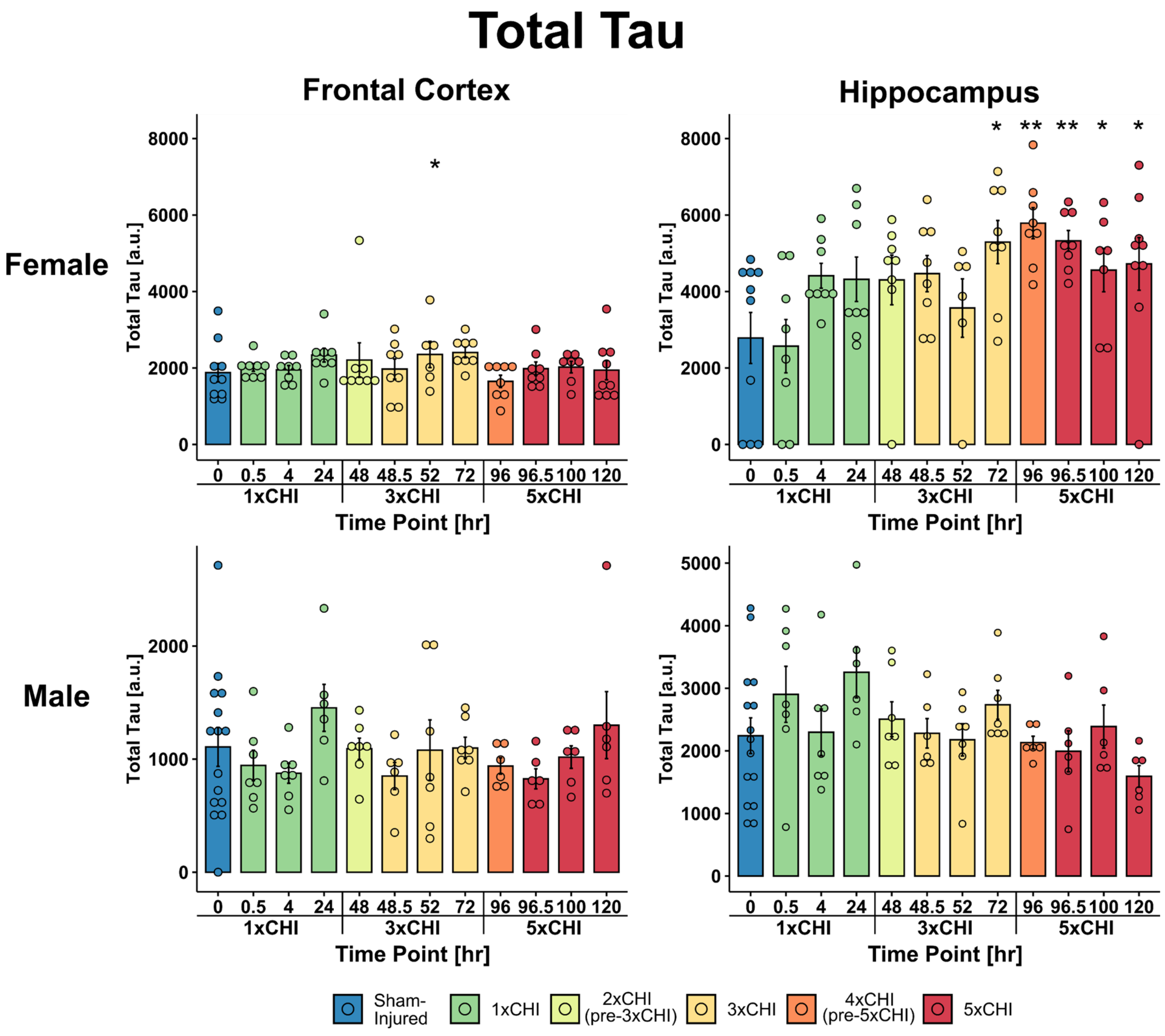

Supplementary Figure S9. Total tau by sex and brain region. Total tau was measured by Luminex ELISA (Millipore Sigma HNABTMAG-68K) in male and female frontal cortex and hippocampus tissue (*p<0.05, **p<0.01, Wilcox test vs sham-injured) (mean ± SEM). Male and female samples run separately.

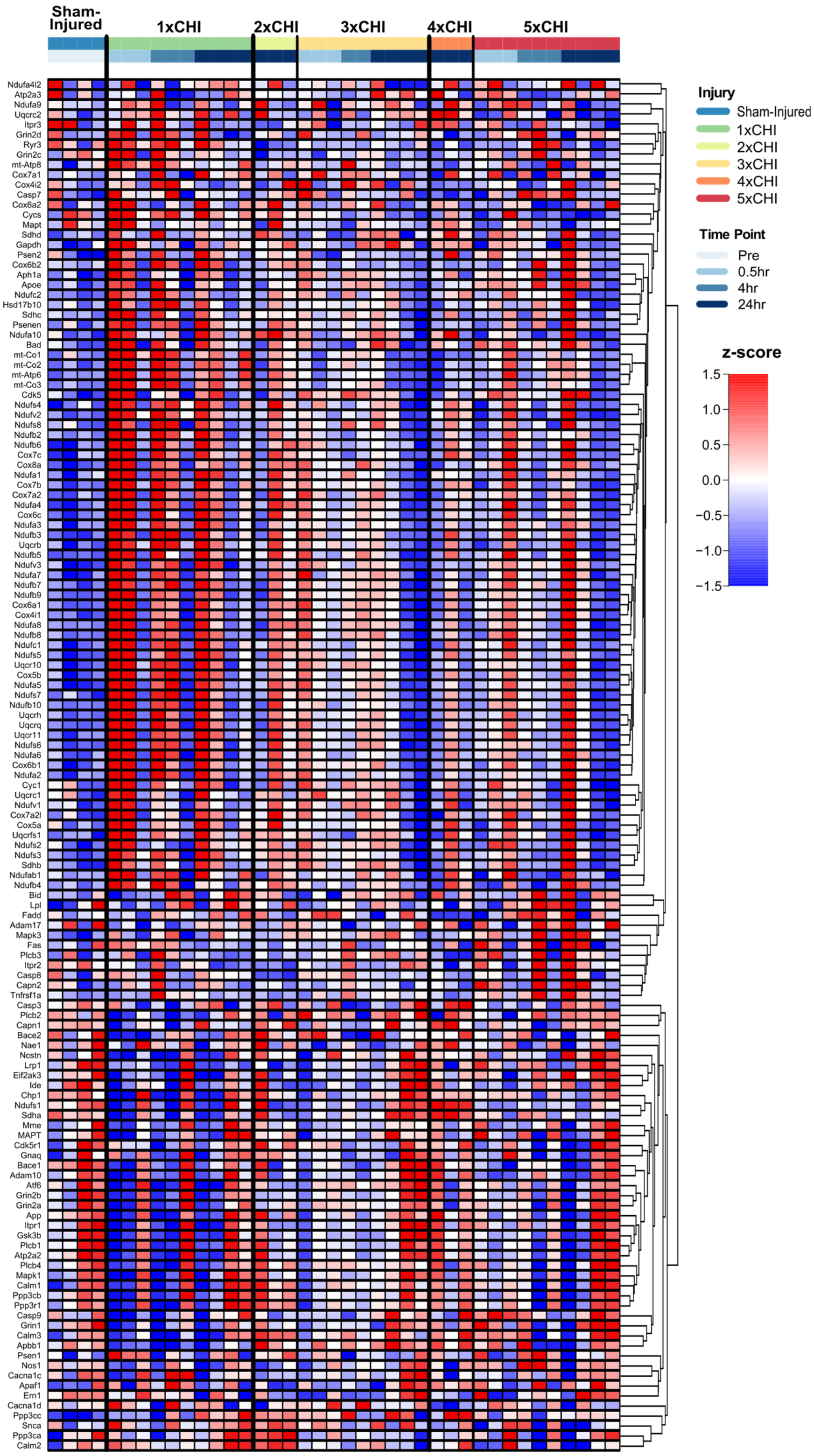

Supplementary Figure S10. Kyoto Encyclopedia of Genes and Genomes (KEGG) Alzheimer's disease gene set. RNAseq data from 204mo female 3xTg-AD somato-motor cortex lysates (n=2-4). Heatmap shows subset of all expressed genes from the KEGG Alzheimer's disease gene set (MSigDB C2 collection). RNAseq data is normalized, variance stabilized (**Methods**), then z-scored within each gene (rows) across all animals (columns). Genes are clustered by unweighted pair group method with arithmetic mean using Euclidean distance.

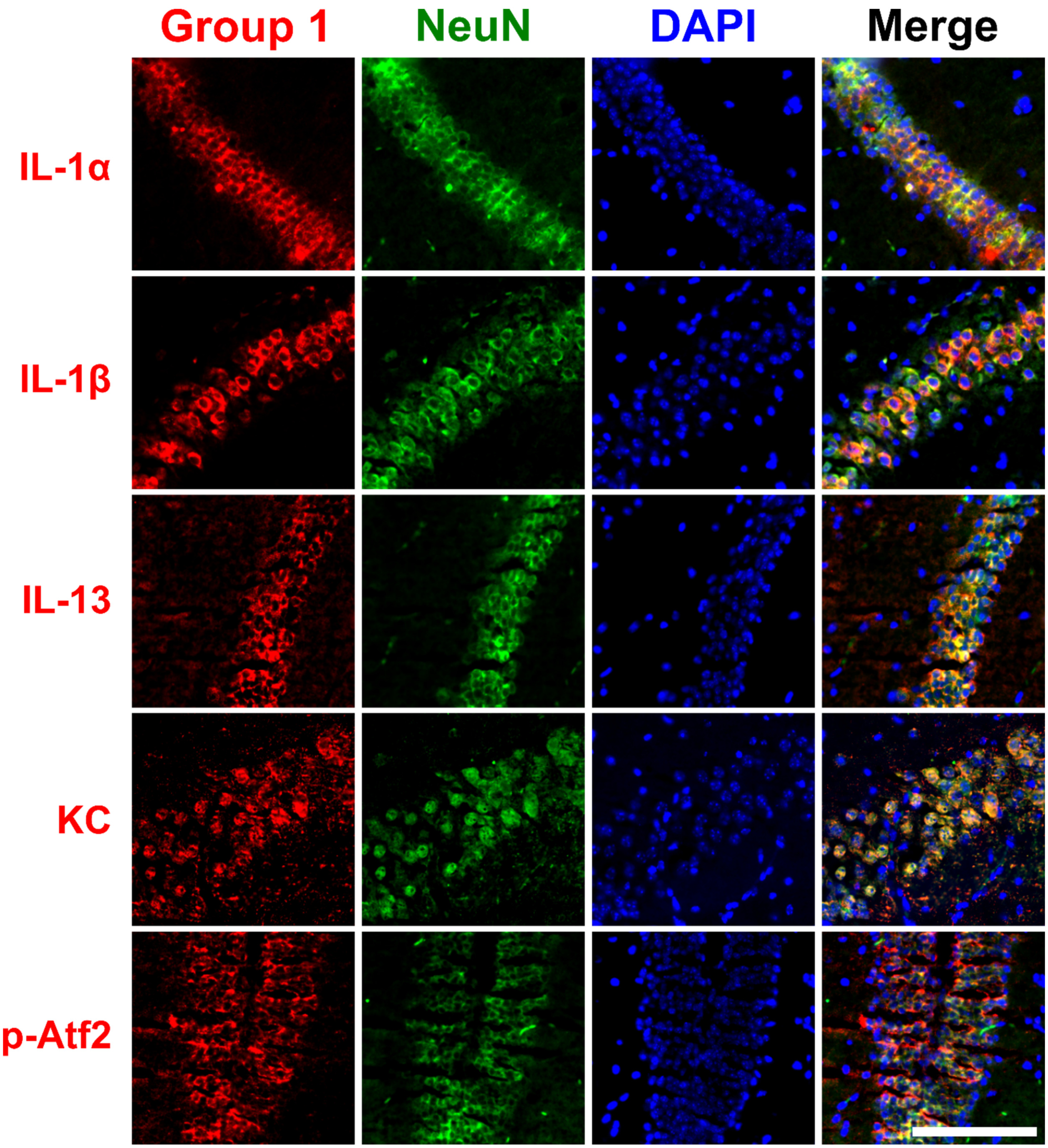

Supplementary Figure S11. Group 1 cytokines and phospho-proteins co-label with NeuN in hippocampus 24hr post-CHI. Group 1 cytokines (IL-1α, IL-1β, IL-13, and KC) and MAPK phospho-protein phospho-Atf2 (red) co-stained with NeuN (green) and DAPI (blue) show extensive neuronal localization in the hippocampus 24hr post-CHI in female 3xTg-AD mice aged 2-4mo (scale bar: 500µm, representative section from n=6 mice 24hr post-1xCHI).

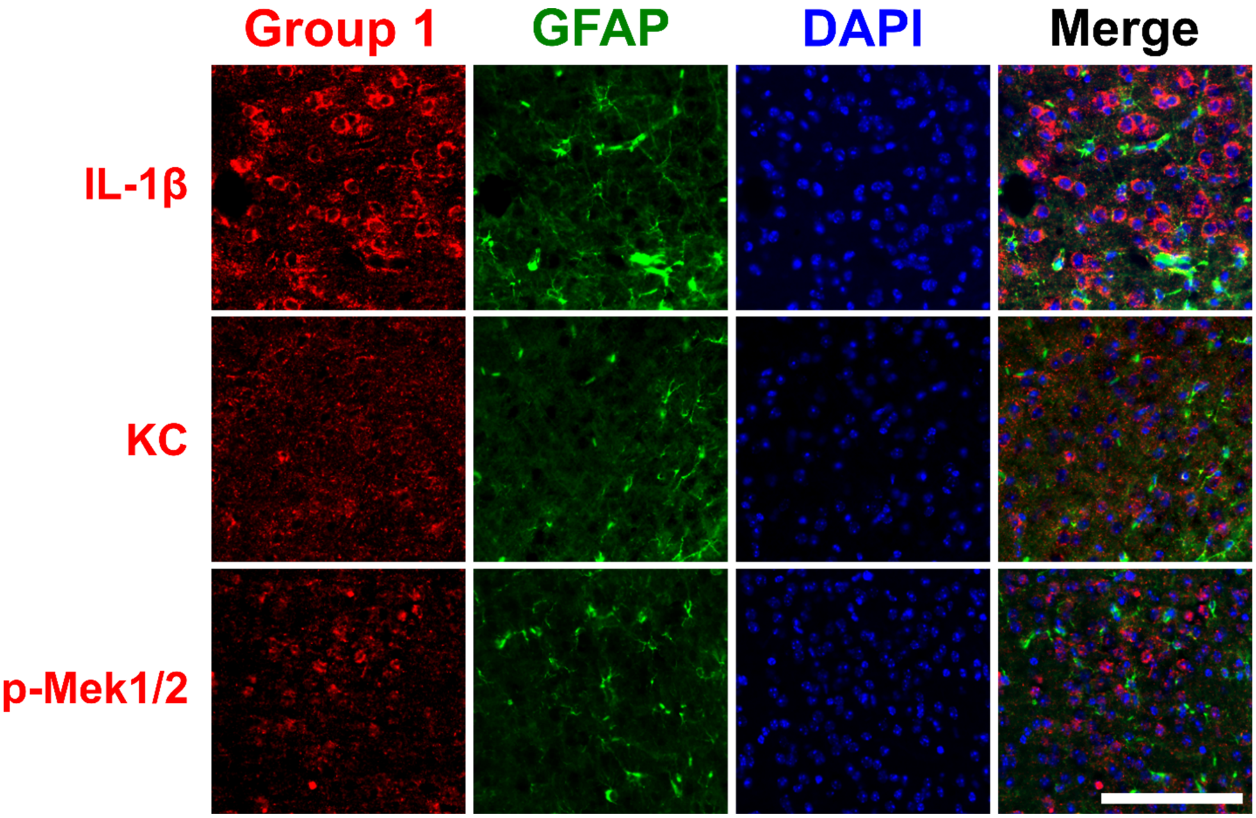

Supplementary Figure S12. Group 1 cytokines and phospho-proteins do not co-label with astrocyte marker GFAP in the cortex 24hr post-CHI. Group 1 cytokines (IL-1β and KC) and MAPK phospho-protein phospho-Mek1/2 (red) co-stained with GFAP (green) and DAPI (blue) showed minimal astrocyte co-label in the cortex 24hr post-CHI in female 3xTg-AD mice aged 2-4mo (scale bar: 500μm).

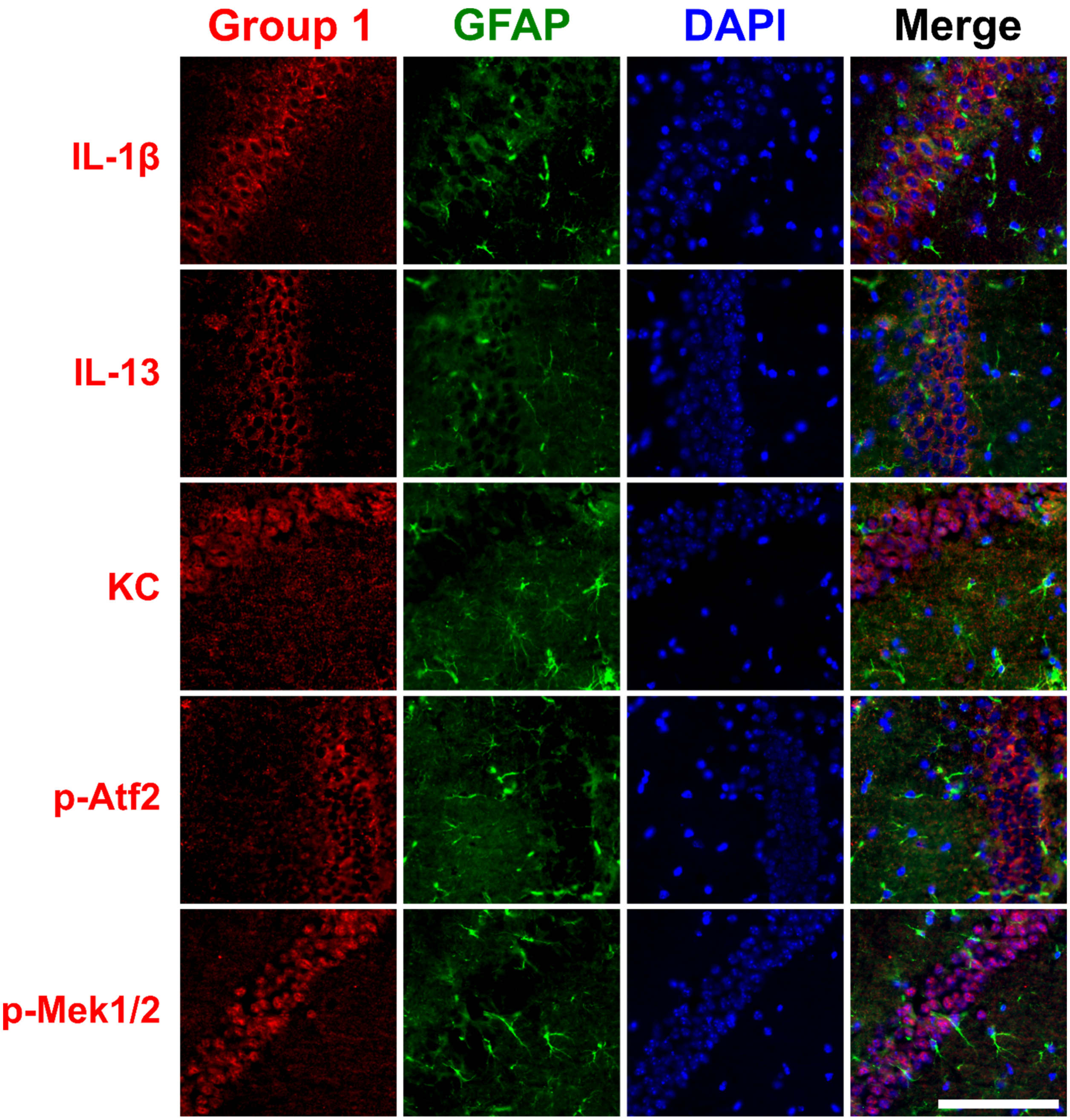

Supplementary Figure S13. Group 1 cytokines and phospho-proteins do not co-label with astrocyte marker GFAP in the hippocampus 24hr post-CHI. Group 1 cytokines (IL-1β, IL-13, and KC) and MAPK phospho-proteins (phospho-Atf2 and phospho-Mek1/2) (red) co-stained with GFAP (green) and DAPI (blue) showed minimal astrocyte co-label in the hippocampus 24hr post-CHI in female 3xTg-AD mice aged 2-4mo (scale bar: 500µm).

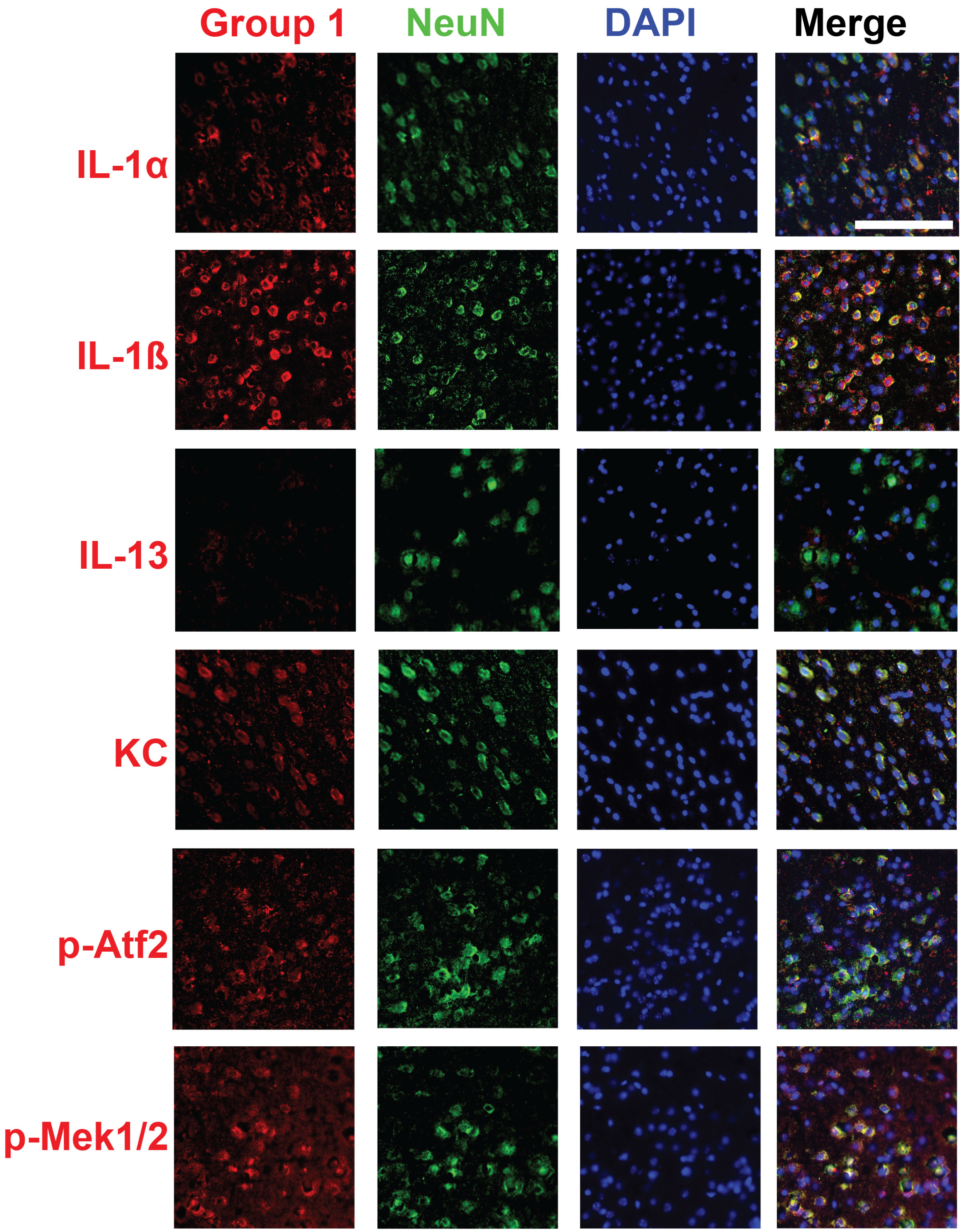

Supplementary Figure S14. Group 1 cytokines and phospho-proteins co-label with NeuN in 3xTg-AD frontal cortex of sham-injured animals. Group 1 cytokines (IL-1α, IL-1β, IL-13, and KC) and MAPK phospho-proteins (phospho-Atf2 and phospho-Mek1/2) (red) co-stained with NeuN (green) and DAPI (blue) show neuronal localization in the 3xTg-AD frontal cortex in sham-injured female 3xTg-AD mice aged 2-4mo (scale bar: 500µm, representative from n=6 sham-injured animals).

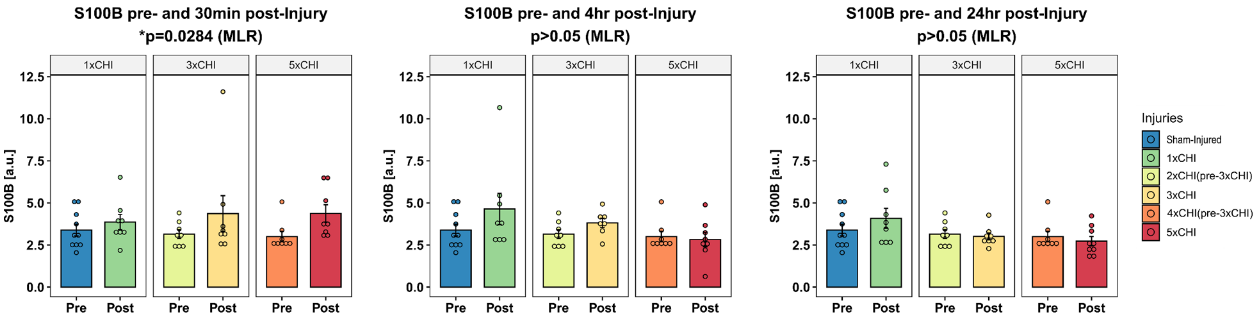

Supplementary Figure S15. Cortical astrocytes become reactive following mild TBI: S100B. Cortical S100 calcium-binding protein B (S100B) is significantly upregulated 30min post-injury vs pre-injury but not 4hr or 24hr post-injury (*p<0.05; multiple linear regression model, pre/post and number of injuries) (mean ± SEM).

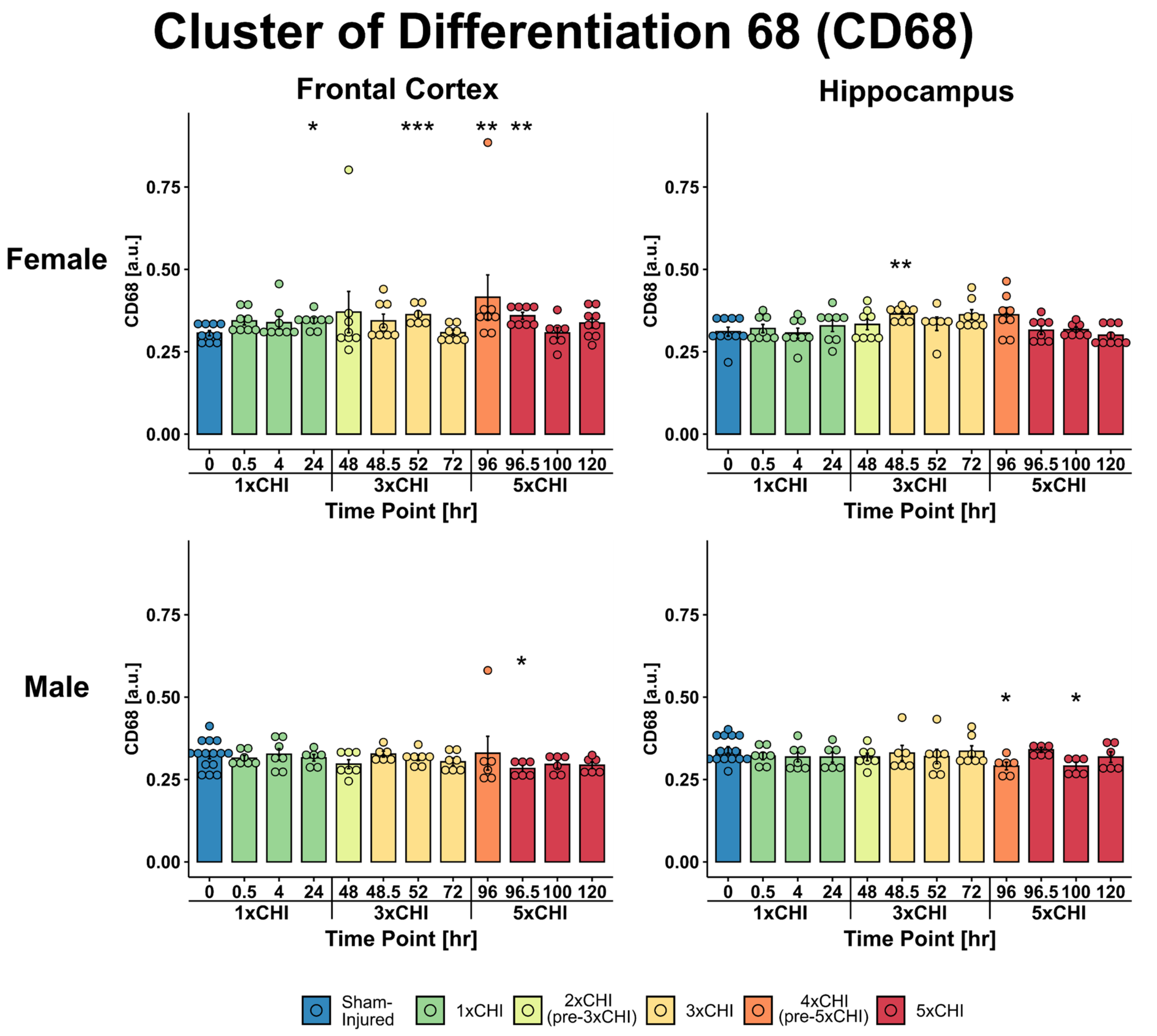

Supplementary Figure S16. CD68 was measured by ELISA in male and female frontal cortex and hippocampus tissue (*p<0.05, **p<0.01, ***p<0.001, Wilcox test vs sham-injured) (mean ± SEM). Male and female samples run simultaneously.
